# Supplementary material for: Clinical interventions for adults with comorbid alcohol use and depressive disorders: A systematic review and network meta-analysis
Source: PLoS Med. 2021 Oct 8;18(10):e1003822. doi: 10.1371/journal.pmed.1003822 (PMC8535380; doi:10.1371/journal.pmed.1003822)
Supplement: S1 Text — (DOCX) [file pmed.1003822.s004.docx]

**S1 Text. Supplementary Online Content**

**eMethods 1**. Detailed Procedures for Systematic Review and Network Meta-Analysis

**eMethods 2**. Electronic Search Strategies

**eMethods 3**. Eligible Diagnoses

**eMethods 4**. List of included studies.

**eMethods 5**. Link to Open Science Framework page with RMarkdown code and HTML output

**eTable 1**. Summary of Studies Included in the Pharmacological Intervention Network

**eTable 2**. Summary of Studies Included in the Psychological Intervention Network

**eTable 3**. Summary of Outcome Data Across Studies

**eTable 4.** Summary of Risks of Bias Across Studies

**eTable 5.** Pharmacological Intervention Effect Estimates at Post-Intervention for Remission from Depression and Alcohol Use

**eTable 6.** Pharmacological Intervention Effect Estimates at Post-Intervention for Depressive and Withdrawal/Craving Symptoms

**eTable 7.** Pharmacological Intervention Effect Estimates at Post-Intervention for Drinking

**eTable 8.** Pharmacological Intervention Effect Estimates at Post-Intervention for Health-Related Quality of Life and Functional Status

**eTable 9.** Pharmacological Intervention Effect Estimates at Post-Intervention for Adverse Events

**eFigure 1**. Forest Plot of Effect Estimates (Active Pharmacological Interventions versus Pharmacological Placebo) on Remission from Depression at Post-Intervention

**eFigure 2.** Forest Plot of Effect Estimates (Active Pharmacological Interventions versus Pharmacological Placebo) on Remission from Alcohol Use at Post-Intervention

**eFigure 3.** Forest Plot of Effect Estimates (Active Pharmacological Interventions versus Pharmacological Placebo) on Depressive Symptoms at Post-Intervention

**eFigure 4.** Forest Plot of Effect Estimates (Active Pharmacological Interventions versus Pharmacological Placebo) on Withdrawal/Craving Symptoms at Post-Intervention

**eFigure 5.** Forest Plot of Effect Estimates (Active Pharmacological Interventions versus Pharmacological Placebo) on Alcohol Use at Post-Intervention

**eFigure 6.** Forest Plot of Effect Estimates (Active Pharmacological Interventions versus Pharmacological Placebo) on Heavy Drinking at Post-Intervention

**eFigure 7.** Forest Plot of Effect Estimates (Active Pharmacological Interventions versus Pharmacological Placebo) on Functional Status at Post-Intervention

**eFigure 8.** Forest Plot of Effect Estimates (Active Pharmacological Interventions versus Pharmacological Placebo) on Adverse Events at Post-Intervention

**eFigure 9.** Forest Plot of Effect Estimates (Active Pharmacological Interventions versus Pharmacological Placebo) on Serious Adverse Events at Post-Intervention

**eFigure 10.** Pharmacologic Network Geometry of Remission from Depression

**eFigure 11.** Pharmacologic Network Geometry of Remission from Alcohol

**eFigure 12.** Psychological Network Geometry of Remission from Alcohol

**eFigure 13.** Pharmacologic Network Geometry of Depressive Symptoms

**eFigure 14.** Psychological Network Geometry of Depressive Symptoms

**eFigure 15.** Pharmacologic Network Geometry of Alcohol Use

**eFigure 16.** Psychological Network Geometry of Alcohol Use

**eFigure 17.** Pharmacologic Network Geometry of Heavy Drinking

**eFigure 18.** Pharmacologic Network Geometry of Withdrawal/Craving Symptoms

**eFigure 19.** Psychological Network Geometry of Withdrawal/Craving Symptoms

**eFigure 20.** Pharmacologic Network Geometry of Functional Status

**eFigure 21.** Pharmacologic Network Geometry of Adverse Events

**eFigure 22.** Pharmacologic Network Geometry of Serious Adverse Events

**eReferences**

**eMethods 1**. Detailed Procedures for Systematic Review and Network Meta-Analysis.

We performed a systematic review to identify RCTs testing the effects of clinical interventions for treating individuals with co-occurring alcohol use disorders (AUDs) and depressive disorders, in order to conduct a network meta-analysis (NMA) to determine the comparative effectiveness of interventions. We registered the protocol for this review in PROSPERO (CRD42017078239), the international prospective register of systematic reviews, prior to completing formal screening of search results against eligibility criteria ([https://www.crd.york.ac.uk/prospero/display_record. php?RecordID=78239)](https://www.crd.york.ac.uk/prospero/display_record.%20php?RecordID=78239)). We prepared the protocol and this report using the relevant Preferred Reporting Items for Systematic Reviews and Meta-Analyses (PRISMA) Statements [1-3], as well as the Methodological Expectations of Cochrane Intervention Reviews (MECIR) [4].

**Search strategy**

We searched for RCTs in the following electronic databases through December 1, 2020: CINAHL, Cochrane Database of Systematic Reviews (CDSR), EMBASE, PubMed, PsycINFO, and Web of Science. In addition, we searched the clinical trial registries ClinicalTrials.gov, Cochrane Central Register of Controlled Trials (CENTRAL), and the World Health Organization (WHO) International Clinical Trials Registry Platform (ICTRP). A Reference Librarian for RAND’s Knowledge Services (JL) developed the search strings in consultation with the lead and senior authors (SG and SH) using terms identified in previous reviews on interventions for AUDs and depressive disorders [5-11]. We used searched terms related to alcohol use, depression, and randomized trials. We also reference-mined the bibliographies of previous systematic reviews.

**Eligibility criteria**

***Studies***

We applied the following inclusion and exclusion criteria related to the types of participants, interventions, comparators, outcomes, timings, settings, and study designs (PICOTSS):

- Participants: We included studies with adult participants (i.e., at least 50% of the sample was 18 years of age or older) who had a clinical diagnosis of both an AUD and depressive disorder according to DSM or ICD diagnostic criteria. We included studies with participants who have additional comorbidities (e.g., other mental health or substance use disorders) so long as the patients had a clinical diagnosis of both an AUD and depressive disorder. In addition to formal diagnostic procedures, we also included studies that used non-operationalized diagnostic criteria, validated clinician-reported symptom questionnaires, or self-reported symptom questionnaires that used established thresholds to identify patients as explicitly having eligible diagnoses.
- Interventions: We included studies evaluating a clinical intervention to treat patients with AUD and depressive disorder. We did not exclude studies based on the specific therapeutic approach (e.g., psychotherapies, pharmacotherapies, complementary and alternative medicine) so long as the evaluated intervention was intended to improve depressive symptoms or reduce alcohol use.
- Comparators: We included studies with any comparator (active comparator, "treatment as usual" or "standard care," placebo, wait-list control, no-treatment).
- Outcomes: We included studies in meta-analyses that reported one or more of the following outcomes: remission from depression and alcohol use (primary outcomes), depressive symptoms, alcohol use, heavy drinking, withdrawal/craving symptoms, health-related quality of life, functional status, and adverse events.
- Timing: We included studies with any follow-up period for outcome assessment and any intervention dosage (intensity, frequency, duration).
- Setting: We did not exclude studies by setting.
- Study Design: We included parallel group, individually- or cluster-randomized controlled trials (RCTs) only. We did not exclude studies based on publication status (e.g., grey literature, conference abstracts). While we did not restrict studies based on language, we used search limits for English-language reports in our electronic search strategies.

***Classes of intervention nodes***

The term “network” meta-analysis comes from visualizing the interventions that have been evaluated for a population of interest as forming a network in which the interventions are represented by dots (or “nodes”) and comparisons between interventions are represented by lines (or “edges”) in a diagram [12]. We a priori registered the following classes of intervention as the “nodes” of interest for our network:

- Antidepressant pharmacotherapies
  - Examples include sertraline, fluoxetine, and nefazodone.
- Alcohol deterrent pharmacotherapies
  - Examples include acamprosate, naltrexone, and disulfiram.
- Combined pharmacotherapy for both depressive disorder and AUD
  - Examples would include a combination of the above-listed pharmacotherapies for depression and alcohol.
- Cognitive behavioral therapy without pharmacotherapy
  - CBT would include any intervention that explicitly attempts to change patterns of thinking and behavior thought to influence patients’ alcohol- and depressive-related problems and behaviors [13].
- Cognitive behavioral therapy with pharmacotherapy
  - Examples would include a combination of CBT as described above and any above-listed pharmacotherapy.
- Self-management support interventions
  - An example is supportive text-messaging [14].
- Facilitated 12-step interventions
  - Facilitated 12-Step interventions differ from the Alcoholics Anonymous program, which is peer-based and not facilitated [15].
- Pharmacologic placebo
  - An example is a sugar pill with identical look, feel, weight, color, and taste to the active pharmacotherapy.
- Psychological placebo
  - An example is an attention-matched control group.
- Wait-list
  - A wait-list involves a group of participants assigned to receive an intervention after the active intervention group.
- No treatment
  - We assigned interventions to this node when participants did not receive any treatment additional to TAU.
- Other approaches
  - We included interventions from eligible studies when they did not fit into one of the aforementioned a priori nodes.

After we completed the intervention search but prior to extracting and analyzing outcome data, we used the above nodes as a guide in deciding on the final treatment nodes based on consensus among the review team leads (SG and SH), external advisors, and client representatives about the class of each included intervention [16].

**Eligibility screening and assessment**

Two reviewers (the lead author (SG), a doctoral-level experienced systematic reviewer, and one of two doctoral students (GA, EH) with experience in systematic reviews) independently screened titles and abstracts of retrieved citations. We obtained citations judged as potentially eligible by at least one reviewer for independent, full-text screening by the two reviewers. Following a pilot session to ensure similar interpretation of each inclusion and exclusion criterion, the two reviewers independently applied the eligibility criteria to full-texts of citations passing the title and abstract screening stage; we resolved any disagreements between the two reviewers through discussion within the review team. We documented the flow of citations throughout this process in an electronic database (DistillerSR), with reasons for exclusion of citations recorded in the electronic database.

**Data extraction**

The lead author (SG) designed the data collection form with input from the project team. We collected study-level data for each PICOTSS criterion using: the PRISMA-Equity Extension for participant data [17, 18], the template for intervention description and replication (TIDieR) checklist for intervention and comparator data [19], ClinicalTrials.gov criteria for outcomes and timing data [20], the Consolidated Framework for Implementation Research (CFIR) for study setting data [21], and information related to risk of bias for study design [22]:

- Participants: gender, age, race/ethnicity, socioeconomic status, baseline depression symptoms, baseline alcohol consumption, and other comorbid psychological/behavioral health conditions.
- Interventions and Comparators: name, hypothesized mechanism of action, materials, procedures, providers, format/modality, location/timing, amount, pre-planned tailoring or adaptation, post-hoc modifications, fidelity measurement, fidelity data, and co-interventions.
- Outcomes (remission from depression and alcohol use, depressive symptoms, alcohol use, heavy drinking, withdrawal/craving symptoms, health-related quality of life, functional status, and adverse events in adults with a co-occurring AUD and depressive disorder) for each follow-up point of measurement: domain, measure (including psychometric properties), metric (e.g., change from baseline), and method of aggregation (e.g., means, proportions).
- Timing: time-points of outcome assessment.
- Setting: month/year, geographic region, number of sites, inner setting (immediate health care setting where intervention is implemented), and outer setting (external context or environment that might influence intervention implementation).
- Study design: purpose, recruitment method, inclusion and exclusion criteria, starting and ending sample size, items relevant to risk of bias (see “Risk of bias” section below), and whether a power calculation was reported by study authors.

Two reviewers (SG and either GA or EH) independently extracted study-level descriptive data in an electronic database. In addition, the lead author (SG) or biostatistician (MB) extracted all outcome data for meta-analyses. If different reports appeared to be from the same study, we compared descriptions of participants to ensure that data from the same study populations entered each analysis only once.

**Risk of bias within individual studies**

Two reviewers (SG and either GA or EH) independently assessed the risk of bias of included studies using the revised Cochrane Risk of Bias tool [23]. Specifically, reviewers assessed risks of bias related to the following: random sequence generation (selection bias), allocation concealment (selection bias), blinding of participants and providers (performance bias), blinding of outcome assessors (detection bias), completeness of reporting outcome data (attrition bias), selective outcome reporting (reporting bias), and other biases (e.g., implementation fidelity).

**Data synthesis**

We conducted pairwise meta-analyses of all direct comparisons to assess the statistical heterogeneity within each comparison. Then, we qualitatively examined the distribution of characteristics across studies in each network that may modify intervention effects to assess the transitivity assumption of NMA—i.e., that participants hypothetically could be randomized to any interventions included in a network [24]. This transitivity assumption involves assuming that sets of studies comparing different interventions in a network are sufficiently similar to each other with respect to characteristics that moderate their relative effects of interventions, and this assumption leads to assessments of consistency of direct and indirect evidence within each network [12]. Following these assessments, we conducted network meta-analysis (NMA) using random effects models in a frequentist framework with the netmeta package (version 0.9-8) in the R statistical environment [25]. We first grouped outcome data into different follow-up periods: immediately post-intervention, short-term follow-up (1-5 months post-intervention), long-term follow-up (6-11 months post-intervention), and very long-term follow-up (12+ months post-intervention). When individual studies provided multiple eligible outcomes for the same domain and time period, we included the outcomes most similar to outcomes for other studies in the primary analysis in order to reduce statistical heterogeneity. For adverse events, we prioritized outcome conditions in the following order: proportion who experienced an adverse event, mean number of adverse events, and proportion who dropped out due to adverse events. We conducted analyses using alternative outcome data reported in included studies to assess the robustness of findings from the primary analysis.

To explore the geometry of networks in each analysis, we created network diagrams in which each node represents one of the intervention classes, each line connecting two nodes represents that an RCT directly compared the two intervention classes represented by the nodes, and the size of each line represents the number of RCTs directly comparing the two intervention classes represented by the nodes (note that the distance between nodes does not represent any parameters from the data). For each combination of pairwise comparison, outcome, and time point, we used standardized mean differences (SMDs) for continuous outcomes and odds ratios (OR) for dichotomous outcomes, with a 95% confidence interval (95% CI), to estimate intervention effects. We used SMDs rather than mean differences, as we expected studies to use various measures, and we wanted to increase the number of studies that could be combined in a meta-analysis. We coded outcomes so that SMDs < 0 and ORs < 1 corresponded to beneficial effects of the first intervention compared with the second. For each outcome and time point, we ranked interventions in order of effectiveness using p-scores—a frequentist measure comparable to the surface under the cumulative ranking curve (SUCRA) value [26]. P-scores measure the extent of certainty that an intervention is better than another intervention averaged over all competing interventions [27]. They are based on the point estimates and standard errors of network estimates, and their values represent the rank of a treatment within the given range of competing treatment; these values vary from 0 to 1, with 1 meaning an intervention is theoretically the best and 0 meaning an intervention is theoretically the worst.

We addressed within-study correlation of effects from multi-arm trials through the netmeta procedures for reweighting all comparisons of each multi-arm trial [28, 29]. We assumed a constant heterogeneity variance across all comparisons in each network, defined via a generalized methods-of-moments estimate of the between-studies variance [30]. We assessed between-study clinical and methodological heterogeneity by examining characteristics of participants, interventions, outcomes, settings, and study designs of included trials, and we explored important sources in pre-specified sub-group analyses (see below). We assessed between-study statistical heterogeneity for each pairwise comparison using the I^2^ statistic. We assessed transitivity (similar distribution of potential effect modifiers across studies) by systematically tabulating and examining characteristics across trials [24]. We assessed local inconsistency (agreement of direct and indirect evidence in each network) by splitting and comparing direct and indirect evidence [31], and we assessed global inconsistency using design-based decomposition of Cochran’s Q as well as a net heat plot [32]. The net heat plot uses color-coding to highlight hot spots of inconsistency between specific direct evidence in the whole network: the stronger the intensity of the color, the greater the evidence of inconsistency [32]. All techniques for assessing local and global inconsistency require a closed loop, as they require direct and indirect evidence to exist in order for these types of evidence to be compared. We also conducted sensitivity analyses excluding pharmacological interventions that do not have legal approval to be prescribed in the United States, using alternative outcome data reported in included studies, and based on risk of bias assessments.

**Confidence in estimates of intervention effects**

We assessed and reported our confidence in each pairwise effect estimate as well as relative rankings of identified interventions using the Grading of Recommendations Assessment, Development, and Evaluation (GRADE) approach [33-36]. The traditional approach involves initially assigning a body of direct evidence of RCTs a rating of “high” confidence and then assessing five domains for possible downgrading of confidence by one or two levels [34]:

- Limitations of included studies (none, serious, or very serious): we considered downgrading one level (“serious”) when most information is from studies at moderate risk of bias and two levels (“very serious”) from studies at high risk of bias [37].
- Indirectness (none, serious, or very serious): we considered downgrading one level (“serious”) when some differences and two levels (“very serious”) when substantial differences exist between the population, the intervention, or the outcomes measured in relevant research studies and those under consideration in our review [38].
- Inconsistency (none, serious, or very serious): we considered downgrading one level (“serious”) when substantial heterogeneity existed or when only two studies provided information to a meta-analytic estimate, and two levels (“very serious”) when considerable heterogeneity existed or when only one study provided information to a meta-analytic estimate [39].
- Imprecision (none, serious, or very serious): we considered downgrading one level (“serious”) when the 95% CI included the null effect and two levels (“very serious”) when the 95% CI included appreciable benefit or harm [40].
- Publication bias (suspected or undetected): we considered downgrading one level (“suspected”) when evidence suggested a selective publication of study findings that likely substantially alters estimates of a non-null effect [41].

For pairwise estimates in a *network* meta-analysis, rating confidence in indirect evidence for an effect estimate involved taking the lowest confidence rating from effect estimates with a common comparator and assessing whether to downgrade for potential intransitivity. The process further involves: (1) presenting the direct and indirect effect estimates for the pairwise comparison, (2) rating confidence in both estimates, (3) presenting the network estimate for the pairwise comparison, and (4) rating the confidence of the network estimate based on the ratings of the direct and indirect estimates as well as an assessment of coherence [33, 35]. Based on these assessments, we reported our confidence in each pairwise effect estimate and overall ranking using one of four categories [42]:

- **High** indicates that we are very confident that there is a non-null effect—that a pairwise effect estimate indicates that one intervention is beneficial over (superior to) another—or that the intervention ranking is accurate.
- **Moderate** indicates that the review authors are moderately confident that there is a non-null effect or that the intervention ranking is accurate.
- **Low** indicates that the review authors have limited confidence that there is a non-null effect or that the intervention ranking is accurate.
- **Very low** indicates that the review authors have very little confidence that there is a non-null effect or that the intervention ranking is accurate.

A proposed (but unofficial) process for applying GRADE to intervention rankings in a network meta-analysis involves identifying the ranking of each intervention according to p-scores as well as the confidence in the effect estimate of each intervention class versus a referent intervention class.

**eMethods 2**. Electronic Search Strategies

***Original Search Strategy***

**CDSR**

Run: 22 May 2018

"alcohol use disorder*" OR alcoholic OR alcoholics OR alcoholism OR "alcohol related disorder*" OR "binge drink*" OR "alcohol withdrawal*" OR "drinking problem*" OR "alcohol abuse*" OR "alcohol use*" OR "alcohol dependent" OR "alcohol dependence" OR "alcohol disorder" OR "alcohol disorder"

AND

depressive* OR depression OR depressed OR melancholia OR melancholy OR melancholic OR "premenstrual dysphoric disorder*" OR "seasonal affective disorder*" OR dysthymi* OR "mood dysregulation"

**CENTRAL**

Run: 22 May 2018

Alcohol* OR "binge drinking" OR alcoholic OR "drinking problem"
AND
depression OR depressive* OR dysthymia OR melancholy OR dysthymic OR melancholia OR "seasonal affective disorder*" OR "premenstrual dysphoric disorder*" OR "mood dysregulation"

**CINAHL**

Search Run: 22 May 2018

(MH "Alcohol-Related Disorders+") OR TI "alcohol use disorder*" OR AB "alcohol use disorder*" OR TI alcoholic OR AB alcoholic OR TI alcoholics OR AB alcoholics OR TI alcoholism OR AB alcoholism OR TI "alcohol related disorder*" OR AB "alcohol related disorder*" OR TI "binge drink*" OR AB "binge drink*" OR TI "alcohol withdrawal*" OR AB "alcohol withdrawal*" OR TI "drinking problem*" OR AB "drinking problem*" OR TI "alcohol abuse*" OR AB "alcohol abuse*" OR TI "alcohol use*" OR AB "alcohol use*" OR TI "alcohol dependent" OR AB "alcohol dependent" OR TI "alcohol dependence" OR AB "alcohol dependence" OR TI "alcohol disorder" OR AB "alcohol disorder"
AND
(MH "Depression+") OR TI depressive* OR AB depressive* OR TI depression OR AB depression OR TI depressed OR AB depressed OR TI melancholia OR AB melancholia OR TI melancholy OR AB melancholy OR TI melancholic OR AB melancholic OR TI "premenstrual dysphoric disorder*" OR AB "premenstrual dysphoric disorder*" OR TI "seasonal affective disorder*" OR AB "seasonal affective disorder*" OR TI dysthymi* OR AB dysthymi* OR TI "mood dysregulation" OR AB "mood dysregulation"
AND
(MH "Clinical Trials") OR (MH "Randomized Controlled Trials") OR TI randomized OR AB randomized OR TI placebo OR AB placebo OR TI randomly OR AB randomly OR TI trial
NOT
(MH "Animals, Laboratory")
NOT
TI "study protocol*" OR AB "study protocol*" OR TI "trial protocol*" OR AB "trial protocol*"

**ClinicalTrials.gov**

Search Run: 23 May 2018

*Search 1*

((alcohol OR "binge drinking" OR alcoholic OR "drinking problem") AND (depression OR depressive OR dysthymia OR melancholy OR dysthymic OR melancholia OR "seasonal affective disorder" OR "premenstrual dysphoric disorder" OR "mood dysregulation"))

*Search 2*

(alcoholism AND (depression OR depressive OR dysthymia OR melancholy OR dysthymic OR melancholia OR "seasonal affective disorder" OR "premenstrual dysphoric disorder" OR "mood dysregulation"))

**Embase**

Search Run: 22 May 2018

'alcoholism'/exp OR 'Alcohol Use Disorders Identification Test'/exp OR 'alcohol use disorder*':ab,ti OR alcoholic:ab,ti OR alcoholics:ab,ti OR alcoholism:ab,ti OR 'alcohol related disorder*':ab,ti OR 'binge drink*':ab,ti OR 'alcohol withdrawal*':ab,ti OR 'drinking problem*':ab,ti OR 'alcohol abuse*':ab,ti OR 'alcohol use*':ab,ti OR 'alcohol dependent':ab,ti OR 'alcohol dependence':ab,ti OR 'alcohol disorder*':ab,ti

AND
'depression'/exp OR depressive*:ab,ti OR depression:ab,ti OR depressed:ab,ti OR melancholia:ab,ti OR melancholy:ab,ti OR melancholic*:ab,ti OR 'premenstrual dysphoric disorder*':ab,ti OR 'seasonal affective disorder*':ab,ti OR dysthymi*:ab,ti OR 'mood dysregulation':ab,ti
AND
'controlled clinical trial'/exp OR randomized:ab,ti OR placebo:ab,ti OR randomly:ab,ti OR trial:ti
NOT
('animal'/exp NOT 'human'/exp)
NOT
'study protocol*':ab,ti OR 'Trial protocol*':ab,ti

**PsycInfo**

Search Run: 22 May 2018

DE "Alcoholism" OR DE "Alcoholic Psychosis" OR TI "alcohol use disorder*" OR AB "alcohol use disorder*" OR TI alcoholic OR AB alcoholic OR TI alcoholics OR AB alcoholics OR TI alcoholism OR AB alcoholism OR TI "alcohol related disorder*" OR AB "alcohol related disorder*" OR TI "binge drink*" OR AB "binge drink*" OR TI "alcohol withdrawal*" OR AB "alcohol withdrawal*" OR TI "drinking problem*" OR AB "drinking problem*" OR TI "alcohol abuse*" OR AB "alcohol abuse*" OR TI "alcohol use*" OR AB "alcohol use*" OR TI "alcohol dependent" OR AB "alcohol dependent" OR TI "alcohol dependence" OR AB "alcohol dependence" OR TI "alcohol disorder" OR AB "alcohol disorder"
AND
(DE "Major Depression" OR DE "Anaclitic Depression" OR DE "Dysthymic Disorder" OR DE "Endogenous Depression" OR DE "Late Life Depression" OR DE "Postpartum Depression" OR DE "Reactive Depression" OR DE "Recurrent Depression" OR DE "Treatment Resistant Depression") OR (DE "Atypical Depression") OR TI depressive* OR AB depressive* OR TI depression OR AB depression OR TI depressed OR AB depressed OR TI melancholia OR AB melancholia OR TI melancholy OR AB melancholy OR TI melancholic OR AB melancholic OR TI "premenstrual dysphoric disorder*" OR AB "premenstrual dysphoric disorder*" OR TI "seasonal affective disorder*" OR AB "seasonal affective disorder*" OR TI dysthymi* OR AB dysthymi* OR TI "mood dysregulation" OR AB "mood dysregulation"
AND
DE "Clinical Trials" OR TI randomized OR AB randomized OR TI placebo OR AB placebo OR TI randomly OR AB randomly OR TI trial OR MR "Clinical Trial"
NOT
DE "Animal Models"
NOT
TI "study protocol*" OR AB "study protocol*" OR TI "trial protocol*" OR AB "trial protocol*"

**PubMed** **- Search for Clinical Trials**

Search Run: 22 May 2018

“Alcohol related disorders”[MeSH] OR alcohol use disorder*[tiab] OR alcoholic[tiab] OR alcoholics[tiab] OR alcoholism[tiab] OR alcohol related disorder*[tiab] OR binge drink*[tiab] OR alcohol withdrawal*[tiab] OR drinking problem*[tiab] OR alcohol abuse*[tiab] OR alcohol use*[tiab] OR alcohol dependent[tiab]OR alcohol dependence[tiab] OR alcohol disorder*[tiab]
AND
Depressive Disorder”[MeSH] OR “Depression”[MeSH] OR depressive*[tiab] OR depression[tiab] OR depressed[tiab] OR melancholia[tiab] OR melancholy[tiab] OR melancholic*[tiab] OR premenstrual dysphoric disorder*[tiab] OR seasonal affective disorder*[tiab] OR dysthymi*[tiab] OR mood dysregulation[tiab] OR depression[ot] OR depressive[ot]
AND
randomized controlled trial[pt] OR controlled clinical trial[pt] OR randomized[tiab] OR placebo[tiab] OR “clinical trials as topic”[mesh:noexp] OR randomly[tiab] OR trial[ti]
NOT
(“animals”[MeSH] NOT “humans” [MeSH])
NOT
(study protocol*[tiab] OR Trial protocol*[tiab])

**Systematic Reviews Search**

Search Run: 22 May 2018

“Alcohol related disorders”[MeSH] OR alcohol use disorder*[tiab] OR alcoholic[tiab] OR alcoholics[tiab] OR alcoholism[tiab] OR alcohol related disorder*[tiab] OR binge drink*[tiab] OR alcohol withdrawal*[tiab] OR drinking problem*[tiab] OR alcohol abuse*[tiab] OR alcohol use*[tiab] OR alcohol dependent[tiab]OR alcohol dependence[tiab] OR alcohol disorder*[tiab]
AND
“Depressive Disorder”[MeSH] OR “Depression”[MeSH] OR depressive*[tiab] OR depression[tiab] OR depressed[tiab] OR melancholia[tiab] OR melancholy[tiab] OR melancholic*[tiab] OR premenstrual dysphoric disorder*[tiab] OR seasonal affective disorder*[tiab] OR dysthymi*[tiab] OR mood dysregulation[tiab] OR depression[ot] OR depressive[ot]
AND
systematic [sb]
NOT
(“animals”[MeSH] NOT “humans” [MeSH])

**Web of Science**

Search Run: 22 May 2018

TS=("alcohol use disorder*") OR TS=(alcoholic) OR TS=( alcoholics) OR TS=(alcoholism) OR TS=("alcohol related disorder*") OR TS=("binge drink*") OR TS=("alcohol withdrawal*") OR TS=("drinking problem*") OR TS=("alcohol abuse*") OR TS=("alcohol use*") OR TI=("alcohol dependent") OR TS=("alcohol dependence") OR TS=("alcohol disorder")
AND
TS=(depressive*) OR TS=(depression) OR TS=(depressed) OR TS=(melancholia) OR TS=(melancholy) OR TS=(melancholic*) OR TS=("premenstrual dysphoric disorder*") OR TS=("seasonal affective disorder*" ) OR TS=(dysthymi*) OR TS=("mood dysregulation")
AND
TS=(randomized) OR TS=(placebo) OR TS=(randomly) OR TI=(trial)
NOT
TS=(rat OR rats OR mice OR mouse OR monkey OR monkeys))

**WHO ICTRP**

Run: 23 May 2018

*Search 1*

Depress* AND alcohol*

*Search 2*

Binge drink* AND depress*

*Search 3*

Drinking problem AND depress*

*Search 4*

alcohol* AND melanchol*

*Search 5*

alcohol* AND seasonal affective disorder

*Search 6*

alcohol* AND dysthym*

*Search 7*

alcohol* AND premenstrual dysphoric disorder

*Search 8*

alcohol* AND mood dysregulation

*Search 9*

Binge drink* AND melanchol*

*Search 10*

Binge drink* AND seasonal affective disorder

*Search 11*

Binge drink* AND dysthym*

*Search 12*

Binge drink* AND premenstrual dysphoric disorder

*Search 13*

Binge drink* AND mood dysregulation

*Search 14*

Drinking problem AND melanchol*

*Search 15*

Drinking problem AND seasonal affective disorder

*Search 16*

Drinking problem AND dysthym*

*Search 17*

Drinking problem AND premenstrual dysphoric disorder

***Updated Search Strategy***

**PubMed**

***Search Run: 1 December 2020
Coverage: 2018 - present***

***Limits: English Language***

*Search for clinical trials*

“Alcohol related disorders”[MeSH] OR alcohol use disorder*[tiab] OR alcoholic[tiab] OR alcoholics[tiab] OR alcoholism[tiab] OR alcohol related disorder*[tiab] OR binge drink*[tiab] OR alcohol withdrawal*[tiab] OR drinking problem*[tiab] OR alcohol abuse*[tiab] OR alcohol use*[tiab] OR alcohol dependent[tiab]OR alcohol dependence[tiab] OR alcohol disorder*[tiab]
AND
“Depressive Disorder”[MeSH] OR “Depression”[MeSH] OR depressive*[tiab] OR depression[tiab] OR depressed[tiab] OR melancholia[tiab] OR melancholy[tiab] OR melancholic*[tiab] OR premenstrual dysphoric disorder*[tiab] OR seasonal affective disorder*[tiab] OR dysthymi*[tiab] OR mood dysregulation[tiab] OR depression[ot] OR depressive[ot]
AND
randomized controlled trial[pt] OR controlled clinical trial[pt] OR randomized[tiab] OR placebo[tiab] OR “clinical trials as topic”[mesh:noexp] OR randomly[tiab] OR trial[ti]
NOT
(“animals”[MeSH] NOT “humans” [MeSH])
NOT
(study protocol*[tiab] OR Trial protocol*[tiab])

**Results: 171**

*Systematic Reviews Search*

“Alcohol related disorders”[MeSH] OR alcohol use disorder*[tiab] OR alcoholic[tiab] OR alcoholics[tiab] OR alcoholism[tiab] OR alcohol related disorder*[tiab] OR binge drink*[tiab] OR alcohol withdrawal*[tiab] OR drinking problem*[tiab] OR alcohol abuse*[tiab] OR alcohol use*[tiab] OR alcohol dependent[tiab]OR alcohol dependence[tiab] OR alcohol disorder*[tiab]
AND
“Depressive Disorder”[MeSH] OR “Depression”[MeSH] OR depressive*[tiab] OR depression[tiab] OR depressed[tiab] OR melancholia[tiab] OR melancholy[tiab] OR melancholic*[tiab] OR premenstrual dysphoric disorder*[tiab] OR seasonal affective disorder*[tiab] OR dysthymi*[tiab] OR mood dysregulation[tiab] OR depression[ot] OR depressive[ot]

AND
systematic [sb]
NOT
(“animals”[MeSH] NOT “humans” [MeSH])

**PsycInfo**

***Search Run: 1 December 2020
Coverage: 2018-present***

***Limits: English Language; academic journals***

DE "Alcoholism" OR DE "Alcoholic Psychosis" OR TI "alcohol use disorder*" OR AB "alcohol use disorder*" OR TI alcoholic OR AB alcoholic OR TI alcoholics OR AB alcoholics OR TI alcoholism OR AB alcoholism OR TI "alcohol related disorder*" OR AB "alcohol related disorder*" OR TI "binge drink*" OR AB "binge drink*" OR TI "alcohol withdrawal*" OR AB "alcohol withdrawal*" OR TI "drinking problem*" OR AB "drinking problem*" OR TI "alcohol abuse*" OR AB "alcohol abuse*" OR TI "alcohol use*" OR AB "alcohol use*" OR TI "alcohol dependent" OR AB "alcohol dependent" OR TI "alcohol dependence" OR AB "alcohol dependence" OR TI "alcohol disorder" OR AB "alcohol disorder"
AND
(DE "Major Depression" OR DE "Anaclitic Depression" OR DE "Dysthymic Disorder" OR DE "Endogenous Depression" OR DE "Late Life Depression" OR DE "Postpartum Depression" OR DE "Reactive Depression" OR DE "Recurrent Depression" OR DE "Treatment Resistant Depression") OR (DE "Atypical Depression") OR TI depressive* OR AB depressive* OR TI depression OR AB depression OR TI depressed OR AB depressed OR TI melancholia OR AB melancholia OR TI melancholy OR AB melancholy OR TI melancholic OR AB melancholic OR TI "premenstrual dysphoric disorder*" OR AB "premenstrual dysphoric disorder*" OR TI "seasonal affective disorder*" OR AB "seasonal affective disorder*" OR TI dysthymi* OR AB dysthymi* OR TI "mood dysregulation" OR AB "mood dysregulation"
AND
DE "Clinical Trials" OR TI randomized OR AB randomized OR TI placebo OR AB placebo OR TI randomly OR AB randomly OR TI trial OR MR "Clinical Trial"
NOT
DE "Animal Models"
NOT
TI "study protocol*" OR AB "study protocol*" OR TI "trial protocol*" OR AB "trial protocol*"

**CINAHL**

***Search Run: 1 December 2020
Coverage: 2018-present***

***Limits: English Language***

(MH "Alcohol-Related Disorders+") OR TI "alcohol use disorder*" OR AB "alcohol use disorder*" OR TI alcoholic OR AB alcoholic OR TI alcoholics OR AB alcoholics OR TI alcoholism OR AB alcoholism OR TI "alcohol related disorder*" OR AB "alcohol related disorder*" OR TI "binge drink*" OR AB "binge drink*" OR TI "alcohol withdrawal*" OR AB "alcohol withdrawal*" OR TI "drinking problem*" OR AB "drinking problem*" OR TI "alcohol abuse*" OR AB "alcohol abuse*" OR TI "alcohol use*" OR AB "alcohol use*" OR TI "alcohol dependent" OR AB "alcohol dependent" OR TI "alcohol dependence" OR AB "alcohol dependence" OR TI "alcohol disorder" OR AB "alcohol disorder"
AND
(MH "Depression+") OR TI depressive* OR AB depressive* OR TI depression OR AB depression OR TI depressed OR AB depressed OR TI melancholia OR AB melancholia OR TI melancholy OR AB melancholy OR TI melancholic OR AB melancholic OR TI "premenstrual dysphoric disorder*" OR AB "premenstrual dysphoric disorder*" OR TI "seasonal affective disorder*" OR AB "seasonal affective disorder*" OR TI dysthymi* OR AB dysthymi* OR TI "mood dysregulation" OR AB "mood dysregulation"
AND
(MH "Clinical Trials") OR (MH "Randomized Controlled Trials") OR TI randomized OR AB randomized OR TI placebo OR AB placebo OR TI randomly OR AB randomly OR TI trial
NOT
(MH "Animals, Laboratory")
NOT
TI "study protocol*" OR AB "study protocol*" OR TI "trial protocol*" OR AB "trial protocol*"

**Embase**

***Search Run: 1 December 2020
Coverage: 2018-present***

***Limits: English Language; Articles/Reviews/Article in Press***

'alcoholism'/exp OR 'Alcohol Use Disorders Identification Test'/exp OR 'alcohol use disorder*':ab,ti OR alcoholic:ab,ti OR alcoholics:ab,ti OR alcoholism:ab,ti OR 'alcohol related disorder*':ab,ti OR 'binge drink*':ab,ti OR 'alcohol withdrawal*':ab,ti OR 'drinking problem*':ab,ti OR 'alcohol abuse*':ab,ti OR 'alcohol use*':ab,ti OR 'alcohol dependent':ab,ti OR 'alcohol dependence':ab,ti OR 'alcohol disorder*':ab,ti
AND
'depression'/exp OR depressive*:ab,ti OR depression:ab,ti OR depressed:ab,ti OR melancholia:ab,ti OR melancholy:ab,ti OR melancholic*:ab,ti OR 'premenstrual dysphoric disorder*':ab,ti OR 'seasonal affective disorder*':ab,ti OR dysthymi*:ab,ti OR 'mood dysregulation':ab,ti
AND
'controlled clinical trial'/exp OR randomized:ab,ti OR placebo:ab,ti OR randomly:ab,ti OR trial:ti
NOT
('animal'/exp NOT 'human'/exp)
NOT
'study protocol*':ab,ti OR 'Trial protocol*':ab,ti

**Web of Science**

***Search Run: 1 December 2020
Coverage: 2018-present***

***Limits: English Language; article/review/early access***

TS=("alcohol use disorder*") OR TS=(alcoholic) OR TS=( alcoholics) OR TS=(alcoholism) OR TS=("alcohol related disorder*") OR TS=("binge drink*") OR TS=("alcohol withdrawal*") OR TS=("drinking problem*") OR TS=("alcohol abuse*") OR TS=("alcohol use*") OR TI=("alcohol dependent") OR TS=("alcohol dependence") OR TS=("alcohol disorder")
AND
TS=(depressive*) OR TS=(depression) OR TS=(depressed) OR TS=(melancholia) OR TS=(melancholy) OR TS=(melancholic*) OR TS=("premenstrual dysphoric disorder*") OR TS=("seasonal affective disorder*" ) OR TS=(dysthymi*) OR TS=("mood dysregulation")
AND
TS=(randomized) OR TS=(placebo) OR TS=(randomly) OR TI=(trial)
NOT
TS=(rat OR rats OR mice OR mouse OR monkey OR monkeys))

**CENTRAL**

***Run: 1 December 2020
Coverage: 2018- present (NOT including ICTRP or CENTRAL records)***

Alcohol* OR "binge drinking" OR alcoholic OR "drinking problem"
AND
depression OR depressive* OR dysthymia OR melancholy OR dysthymic OR melancholia OR "seasonal affective disorder*" OR "premenstrual dysphoric disorder*" OR "mood dysregulation"

**CDSR**

***Run: 1 December 2020
Coverage: 2018 – present***

("alcohol use disorder*" OR alcoholic OR alcoholics OR alcoholism OR "alcohol related disorder*" OR "binge drink*" OR "alcohol withdrawal*" OR "drinking problem*" OR "alcohol abuse*" OR "alcohol use*" OR "alcohol dependent" OR "alcohol dependence" OR "alcohol disorder" OR "alcohol disorder"):ti,ab,kw
AND

(depressive* OR depression OR depressed OR melancholia OR melancholy OR melancholic OR "premenstrual dysphoric disorder*" OR "seasonal affective disorder*" OR dysthymi* OR "mood dysregulation"):ti,ab,kw

**ClinicalTrials.gov**

***Search Run: 1 December 2020
First posted from 05/22/2018 – 12/1/2020***

*Search 1*

((alcohol OR "binge drinking" OR alcoholic OR "drinking problem") AND (depression OR depressive OR dysthymia OR melancholy OR dysthymic OR melancholia OR "seasonal affective disorder" OR "premenstrual dysphoric disorder" OR "mood dysregulation"))

*Search 2*

(alcoholism AND (depression OR depressive OR dysthymia OR melancholy OR dysthymic OR melancholia OR "seasonal affective disorder" OR "premenstrual dysphoric disorder" OR "mood dysregulation"))

**WHO ICTRP**

***Run: 1 December 2020
Date of registration between 1/1/2018-1/12/2020
General search (exported all and removed any pre-2018)*
*CENTRAL limiting to Source: ICTRP***

Alcohol* OR "binge drinking" OR alcoholic OR "drinking problem"
AND
depression OR depressive* OR dysthymia OR melancholy OR dysthymic OR melancholia OR "seasonal affective disorder*" OR "premenstrual dysphoric disorder*" OR "mood dysregulation"

**eMethods 3**. Eligible Diagnoses

We used the Diagnostic and Statistical Manual (DSM) of Mental Disorders, fifth edition (DSM-V) to generate the below list of diagnoses [43], with the International Classification of Diseases (ICD) codes also provided [44]. We included corresponding diagnoses from previous versions of the DSM as well [45].

**Alcohol use disorder diagnoses**

***Included diagnoses***

- Mild Alcohol Use Disorder
  - ICD-9: 30.500
  - ICD-10: F10.10
- Moderate Alcohol Use Disorder
  - ICD-9: 303.90
  - ICD-10: F10.20
- Severe Alcohol Use Disorder
  - ICD-9: 303.90
  - ICD-10: F10.20
- Alcohol Withdrawal
  - ICD-9: 291.81
  - ICD-10: F10.239, F10.232
- Unspecified Alcohol-Related Disorder
  - ICD-9: 291.9
  - ICD-10: F10.99

***Excluded diagnoses***

- Alcohol Intoxication
  - ICD-9: 303.00
  - ICD-10: F10.929

**Depressive disorder diagnoses**

***Included diagnoses***

- Alcohol-Induced Depressive Disorder
  - ICD-9: 291.89
  - ICD-10: F10.14, F10.24, F10.94
- Major Depressive Disorder in Full Remission (Single Episode)
  - ICD-9: 296.26
  - ICD-10: F32.5
- Major Depressive Disorder in Full Remission (Recurrent Episode)
  - ICD-9: 296.36
  - ICD-10: F33.52
- Major Depressive Disorder in Partial Remission (Single Episode)
  - ICD-9: 296.25
  - ICD-10: F32.4
- Major Depressive Disorder in Partial Remission (Recurrent Episode)
  - ICD-9: 296.35
  - ICD-10: F33.41
- Major Depressive Disorder with Psychotic Features (Single Episode)
  - ICD-9: 296.24
  - ICD-10: F32.3
- Major Depressive Disorder with Psychotic Features (Recurrent Episode)
  - ICD-9: 296.34
  - ICD-10: F33.3
- Mild Major Depressive Disorder (Single Episode)
  - ICD-9: 296.21
  - ICD-10: F32.0
- Mild Major Depressive Disorder (Recurrent Episode)
  - ICD-9: 296.31
  - ICD-10: F33.0
- Moderate Major Depressive Disorder (Single Episode)
  - ICD-9: 296.22
  - ICD-10: F32.1
- Moderate Major Depressive Disorder (Recurrent Episode)
  - ICD-9: 296.32
  - ICD-10: F33.1
- Other Specified Depressive Disorder
  - ICD-9: 311
  - ICD-10: F32.8
- Persistent Depressive Disorder (Dysthymia)
  - ICD-9: 300.4
  - ICD-10: F34.1
- Severe Major Depressive Disorder (Single Episode)
  - ICD-9: 296.23
  - ICD-10: F32.2
- Severe Major Depressive Disorder (Recurrent Episode)
  - ICD-9: 296.33
  - ICD-10: F33.2
- Unspecified Depressive Disorder
  - ICD-9: 311
  - ICD-10: F32.9
- Unspecified Major Depressive Disorder (Single Episode)
  - ICD-9: 296.20
  - ICD-10: F32.9
- Unspecified Major Depressive Disorder (Recurrent Episode)
  - ICD-9: 296.30
  - ICD-10: F33.9

***Excluded diagnoses***

- Bipolar I Disorder: Current or Most Recent Episode Depressed
  - ICD-9: 296.51, 296.52, 296.53, 296.43, 296.55, 296.56, 296.50
  - ICD-10: F31.31, F31.32, F31.4, F31.5, F31.75, F31.76, F31.9
- Bipolar II Disorder
  - ICD-9: 296.89
  - ICD-10: F31.81
- Cyclothymic Disorder
  - ICD-9: 301.13
  - ICD-10: F34.0
- Depressive Disorder due to Another Medical Condition
  - ICD-9: 293.83
  - ICD-10: F06.31, F06.32, F06.34
- Depressive Episodes with Short-Duration Hypomania
- Disruptive Mood Dysregulation Disorder
  - ICD-9: 296.99
  - ICD-10: F34.8
- Other Specified Bipolar and Related Disorder
  - ICD-9: 296.89
  - ICD-10: F31.89
- Premenstrual Dysphoric Disorder
  - ICD-9: 625.4
  - ICD-10: N94.3
- Substance/Medication-Induced Depressive Disorder (other than alcohol)
  - ICD-9: 292.84
  - ICD-10: F11.14, F11.24, F11.94, F13.14, F13.24, F13.94, F14.14, F14.24, F14.94, F15.14, F15.24, F15.94, F16.14, F16.24, F16.94, F18.14, F18.24, F18.94, F19.14, F19.24, F19.94
- Unspecified Bipolar and Related Disorder
  - ICD-9: 296.80
  - ICD-10: F31.9

**eMethods 4**. List of included studies

1. Adamson 2015
2. Agyapong 2012
3. Altamura 1990
4. Altintoprak 2008
5. Butterworth 1971
6. Cocchi 1997
7. Cornelius 1997
8. Cornelius 2016
9. Golik-Gruber 2003
10. Gual 2003
11. Han 2013
12. Hernandez-Avila 2004
13. Holzhauer 2017
14. Kranzler 2006
15. Krupitsky 2013
16. Loo 1988
17. Markowitz 2008
18. Mason 1996
19. McGrath 1996
20. McLean 1986
21. Mielke 1978
22. Moak 2003
23. Muhonen 2008
24. O'Reily 2019
25. Oslin 2005
26. Petersen 2009
27. Pettinati 2010
28. Ralevski 2013
29. Roy 1998
30. Roy-Byrne 2000
31. Salloum 2007
32. Shaw 1975
33. Thapinta 2014
34. Thapinta 2017
35. Witte 2012
36. Zielinski 1979

**Adamson 2015**

***Main reference***

Adamson, S. J., J. D. Sellman, J. A. Foulds, C. M. Frampton, D. Deering, A. Dunn, J. Berks, L. Nixon, and G. Cape, “A Randomized Trial of Combined Citalopram and Naltrexone for Nonabstinent Outpatients with Co-Occurring Alcohol Dependence and Major Depression,” *Journal of Clinical Psychopharmacology,* Vol. 35, No. 2, April 2015, pp. 143–149.

***Additional references***

ACTRN12606000413527, “Treatment Evaluation of Alcohol and Mood,” 2006. As of March 15, 2019: <http://www.anzctr.org.au/ACTRN12606000413527.aspx>

Foulds, J. A., J. Douglas Sellman, S. J. Adamson, J. M. Boden, R. T. Mulder, and P. R. Joyce, “Depression Outcome in Alcohol Dependent Patients: An Evaluation of the Role of Independent and Substance-Induced Depression and Other Predictors,” *Journal of Affective Disorders,* Vol. 174, March 15, 2015, pp. 503–510.

Foulds, J. A., R. T. Mulder, G. Newton-Howes, S. J. Adamson, J. M. Boden, and J. D. Sellman, “Personality Predictors of Drinking Outcomes in Depressed Alcohol-Dependent Patients,” *Alcohol and Alcoholism,* Vol. 51, No. 3, May 2016, pp. 296–301.

Foulds, J. A., K. Ton, M. A. Kennedy, S. J. Adamson, R. T. Mulder, and J. D. Sellman, “OPRM1 Genotype and Naltrexone Response in Depressed Alcohol-Dependent Patients,” *Pharmacogenetic and Genomics,* Vol. 25, No. 5, May 2015, pp. 270–273.

Richardson, D., Adamson, S., Deering, D.. "Therapeutic alliance predicts mood but not alcohol outcome in a comorbid treatment setting," J Subst Abuse Treat. 2018. 91:28-36.

**Agyapong 2012**

***Main reference***

Agyapong, V. I., S. Ahern, D. M. McLoughlin, and C. K. Farren, “Supportive Text Messaging for Depression and Comorbid Alcohol Use Disorder: Single-Blind Randomised Trial,” *Journal of Affective Disorders,* Vol. 141, No. 2–3, December 10, 2012, pp. 168–176.

***Additional references***

Agyapong, V., S. Ahern, and C. Farren, “Supportive Text Messaging for Depression and Comorbid Alcohol Use Disorder: Single-Blind Randomised Trial,” *Alcohol and Alcoholism,* Vol. 48, September 2013, pp. i24.

Agyapong, V., D. McLoughlin, and C. K. Farren, “Usefulness of Supportive Text Messages to Patients with Alcohol Use Disorder and Comorbid Depression—A Single-Blind Randomised Trial,” *European Psychiatry,* Vol. 27, 2012, pp. 1.

Agyapong, V., D. M. McLoughlin, and C. K. Farren, “6-month Outcomes of a Single Blind Randomised Trial of Supportive Text Messaging for Depression and Comorbid Alcohol Use Disorder,” *European Psychiatry,* Vol. 28, 2013.

Agyapong, V. I., D. M. McLoughlin, and C. K. Farren, “Six-Months Outcomes of a Randomised Trial of Supportive Text Messaging for Depression and Comorbid Alcohol Use Disorder,” *Journal of Affective Disorders,* Vol. 151, No. 1, October 2013, pp. 100–104.

Agyapong, V. I., J. Milnes, D. M. McLoughlin, and C. K. Farren, “Perception of Patients with Alcohol Use Disorder and Comorbid Depression About the Usefulness of Supportive Text Messages,” *Technology and Health Care,* Vol. 21, No. 1, 2013, pp. 31–39.

NCT01037868, “Usefulness of Supportive Text Messages in the Treatment of Depressed Alcoholics,” 2009. As of March 18, 2019: <http://clinicaltrials.gov/show/NCT01037868>

**Altamura 1990**

***Main reference***

Altamura, A. C., M. C. Mauri, T. Girardi, and B. Panetta, “Alcoholism and Depression: A Placebo Controlled Study with Viloxazine,” *International Journal of Clinical Pharmacology Research,* Vol. 10, No. 5, 1990, pp. 293–298.

**Altintoprak 2008**

***Main reference***

Altintoprak, A. E., N. Zorlu, H. Coskunol, F. Akdeniz, and G. Kitapcioglu, “Effectiveness and Tolerability of Mirtazapine and Amitriptyline in Alcoholic Patients with Co‐Morbid Depressive Disorder: A Randomized, Double‐Blind Study,” *Human Psychopharmacology: Clinical and Experimental,* Vol. 23, No. 4, 2008, pp. 313–319.

**Butterworth 1971**

***Main reference***

Butterworth, A. T., “Depression Associated with Alcohol Withdrawal: Imipramine Therapy Compared with Placebo,” *Quarterly Journal of Studies on Alcohol,* Vol. 32, No. 2, June 1971, pp. 343–348.

**Cocchi 1997**

***Main reference***

Cocchi, R., “Paroxetine vs Amitryptiline in Depressed Alcoholics,” *European Neuropsychopharmacology,* Vol. 7, Supp. 2, 1997, p. S254.

**Cornelius 1997**

***Main reference***

Cornelius, J. R., I. M. Salloum, J. G. Ehler, P. J. Jarrett, M. D. Cornelius, J. M. Perel, M. E. Thase, and A. Black, “Fluoxetine in Depressed Alcoholics: A Double-Blind, Placebo-Controlled Trial,” *Archives of General Psychiatry,* Vol. 54, No. 8, August 1997, pp. 700–705.

***Additional references***

Cornelius, J. R., K. A. Perkins, I. M. Salloum, M. E. Thase, and H. B. Moss, “Fluoxetine Versus Placebo to Decrease the Smoking of Depressed Alcoholic Patients,” *Journal of Clinical Psychopharmacology,* Vol. 19, No. 2, April 1999, pp. 183–184.

Cornelius, J. R., I. M. Salloum, M. D. Cornelius, J. M. Perel, J. G. Ehler, P. J. Jarrett, R. L. Levin, A. Black, and J. J. Mann, “Preliminary Report: Double-Blind, Placebo-Controlled Study of Fluoxetine in Depressed Alcoholics,” *Psychopharmacology Bulletin,* Vol. 31, No. 2, 1995, pp. 297–303.

Cornelius, J. R., I. M. Salloum, J. G. Ehler, P. J. Jarrett, M. D. Cornelius, A. Black, J. M. Perel, and M. E. Thase, “Double-Blind Fluoxetine in Depressed Alcoholic Smokers,” *Psychopharmacology Bulletin,* Vol. 33, No. 1, 1997, pp. 165–170.

Cornelius, J. R., I. M. Salloum, J. G. Ehler, P. J. Jarrett, J. M. Perel, and M. E. Thase, “Fluoxetine vs Placebo in Depressed Alcoholic Smokers,” *Psychopharmacology Bulletin,* Vol. 32, No. 3, 1996, pp. 424.

Cornelius, J. R., I. M. Salloum, R. F. Haskett, D. C. Daley, M. D. Cornelius, M. E. Thase, and J. M. Perel, “Fluoxetine Versus Placebo in Depressed Alcoholics: A 1-Year Follow-Up Study,” *Addictive Behaviors,* Vol. 25, No. 2, March–April 2000, pp. 307–310.

Cornelius, J. R., I. M. Salloum, R. F. Haskett, J. G. Ehler, P. J. Jarrett, M. E. Thase, and J. M. Perel, “Fluoxetine Versus Placebo for the Marijuana Use of Depressed Alcoholics,” *Addictive Behaviors,* Vol. 24, No. 1, January–February 1999, pp. 111–114.

Cornelius, J. R., I. M. Salloum, M. E. Thase, R. F. Haskett, D. C. Daley, A. Jones-Barlock, C. Upsher, and J. M. Perel, “Fluoxetine Versus Placebo in Depressed Alcoholic Cocaine Abusers,” *Psychopharmacology Bulletin,* Vol. 34, No. 1, 1998, pp. 117–121.

**Cornelius 2016**

***Main reference***

Cornelius, J. R., T. Chung, A. B. Douaihy, L. Kirisci, J. Glance, J. Kmiec, D. FitzGerald, M. A. Wesesky, and I. Salloum, “Mirtazapine in Comorbid Major Depression and an Alcohol Use Disorder: A Double-Blind Placebo-Controlled Pilot Trial,” *Psychiatry Research,* Vol. 242, August 30, 2016, pp. 326–330.

***Additional references***

Cornelius, J., “Two Mirtazapine Pilot Trials in Young and in Older Depressed Alcoholics,” *American Journal on Addictions,* Vol. 25, No. 4, June 2016, pp. 313–314.

Cornelius, J., T. Chung, A. Douaihy, and J. Glance, “Mirtazapine Pilot Trial in Youthful MDD/AUD Subjects,” *American Journal on Addictions,* Vol. 26, No. 3, 2017, pp. 250–251.

Cornelius, J. R., T. A. Chung, A. Douaihy, J. Glance, J. Kmiec, M. A. Wesesky, D. Fitzgerald, and I. Salloum, “Double-Blind Mirtazapine Pilot Trial in AUD/MDD,” *Alcoholism: Clinical and Experimental Research,* Vol. 40, 2016, p. 233A.

Cornelius, J., A. Douaihy, and D. Daley, “Two Mirtazapine Pilot Trials in Depressed Alcoholics,” *American Journal on Addictions,* Vol. 24, No. 1, January 2015, pp. 56–57.

NCT02185131, “Double-Blind Pilot Trial of Mirtazapine for the Treatment of Co-Occurring AD/MDD,” 2014. As of March 18, 2019: https://clinicaltrials.gov/show/NCT02185131

NCT02646449, “Treatment of Young Adults with Comorbid AUD/MDD: A Pilot Medication Trial,” 2016. As of March 18, 2019: https://clinicaltrials.gov/show/NCT02646449

**Golik-Gruber 2003**

***Main reference***

Golik-Gruber, V., D. Breitenfeld, E. N. Gruber, and D. Karlović, “Sulpiride Psychopharmacotherapy in Patients with Alcohol Addiction and Depression Comorbidity,” *Acta Clinica Croatica,* Vol. 42, No. 1, 2003, pp. 29–33.

**Gual 2003**

***Main reference***

Gual, A., M. Balcells, M. Torres, M. Madrigal, T. Diez, and L. Serrano, “Sertraline for the Prevention of Relapse in Detoxicated Alcohol Dependent Patients with a Comorbid Depressive Disorder: A Randomized Controlled Trial,” *Alcohol and Alcoholism,* Vol. 38, No. 6, November–December 2003, pp. 619–625.

**Han 2013**

***Main reference***

Han, D. H., S. M. Kim, J. E. Choi, K. J. Min, and P. F. Renshaw, “Adjunctive Aripiprazole Therapy with Escitalopram in Patients with Co-Morbid Major Depressive Disorder and Alcohol Dependence: Clinical and Neuroimaging Evidence,” *Journal of Psychopharmacology,* Vol. 27, No. 3, 2013, pp. 282–291.

**Hernandez-Avila 2004**

***Main reference***

Hernandez-Avila, C. A., V. Modesto-Lowe, R. Feinn, and H. R. Kranzler, “Nefazodone Treatment of Comorbid Alcohol Dependence and Major Depression,” *Alcoholism: Clinical and Experimental Research,* Vol. 28, No. 3, March 2004, pp. 433–440.

**Holzhauer 2017**

***Main reference***

Holzhauer, C. G., and S. A. Gamble, “Depressive Symptoms Mediate the Relationship Between Changes in Emotion Regulation During Treatment and Abstinence Among Women with Alcohol Use Disorders,” *Psychology of Addictive Behaviors,* Vol. 31, No. 3, May 2017, pp. 284–294.

**Kranzler 2006**

***Main reference***

Kranzler, H. R., T. Mueller, J. Cornelius, H. M. Pettinati, D. Moak, P. R. Martin, R. Anthenelli, K. J. Brower, S. O’Malley, B. J. Mason, D. Hasin, and M. Keller, “Sertraline Treatment of Co-Occurring Alcohol Dependence and Major Depression,” *Journal of Clinical Psychopharmacology,* Vol. 26, No. 1, February 2006, pp. 13–20.

**Krupitsky 2013**

***Main reference***

Krupitsky, E. M., S. M. Yerish, A. S. Kiselev, V. A. Berntsev, N. A. Alexandrovsky, M. N. Torban, S. P. Eroshin, and O. F. Eryshev, “A Double-Blind, Placebo-Controlled, Randomized Clinical Trial of Escitalopram for the Treatment of Affective Disorders in Alcohol Dependent Patients in Early Remission,” in Nash Boutros, ed., *The International Psychiatry and Behavioral Neurosciences Yearbook—2012*: Vol. 2, Hauppauge, N.Y.: Nova Biomedical Books, 2013, pp. 239–256.

***Additional references***

Krupitsky, E. M., S. M. Yerish, V. A. Berntsev, A. S. Kiselev, N. A. Alexandrovsky, M. N. Torban, S. P. Eroshin, and O. F. Eryshev, “Double Blind Placebo Controlled Randomized Clinical Trial of Escitalopram for Alcoholism Comorbid with Affective Disorders (Depression and Anxiety),” *Alcoholism: Clinical and Experimental Research,* Vol. 34, 2010, p. 167A.

Krupitsky, E., S. Yerish, and A. Kiselev, “Clinical Trial of Escitalopram for Alcoholism Comorbid with Affective Disorders,” *European Neuropsychopharmacology,* Vol. 20, 2010, p. S571.

Krupitsky, E., S. Yerish, and A. Kiselev, “Clinical Trial of Escitalopram for Alcoholism Comorbid with Affective Disorders,” *Alcoholism: Clinical and Experimental Research,* Vol. 36, 2012, p. 297A.

**Loo 1988**

***Main reference***

Loo, H., R. Malka, R. Defiance, D. Barrucand, J. Y. Benard, H. Niox-Riviere, A. Raab, A. Sarda, G. Vachonfrance, and A. Kamoun, “Tianeptine and Amitriptyline: Controlled Double-Blind Trial in Depressed Alcoholic Patients,” *Neuropsychobiology,* Vol. 19, No. 2, 1988, pp. 79–85.

**Markowitz 2008**

***Main reference***

Markowitz, J. C., J. H. Kocsis, P. Christos, K. Bleiberg, and A. Carlin, “Pilot Study of Interpersonal Psychotherapy Versus Supportive Psychotherapy for Dysthymic Patients with Secondary Alcohol Abuse or Dependence,” *Journal of Nervous and Mental Disease,* Vol. 196, No. 6, 2008, pp. 468–474.

**Mason 1996**

***Main reference***

Mason, B. J., J. H. Kocsis, E. C. Ritvo, and R. B. Cutler, “A Double-Blind, Placebo-Controlled Trial of Desipramine for Primary Alcohol Dependence Stratified on the Presence or Absence of Major Depression,” *JAMA,* Vol. 275, No. 10, March 13, 1996, pp. 761–767.

***Additional references***

Mason, B. J., and J. H. Kocsis, “Desipramine Treatment of Alcoholism,” *Psychopharmacology Bulletin,* Vol. 27, No. 2, 1991, pp. 155–161.

Mason, B. J., and J. H. Kocsis, “Desipramine in the Treatment of Depressed Alcoholics,” *American Journal of Psychiatry,* Vol. 151, No. 8, August 1994, p. 1248.

**McGrath 1996**

***Main reference***

McGrath, P. J., E. V. Nunes, J. W. Stewart, D. Goldman, V. Agosti, K. Ocepek-Welikson, and F. M. Quitkin, “Imipramine Treatment of Alcoholics with Primary Depression: A Placebo-Controlled Clinical Trial,” *Archives of General Psychiatry,* Vol. 53, No. 3, March 1996, pp. 232–240.

***Additional references***

McGrath, P. J., E .V. Nunes, J. W. Stewart, K. Ocepek-Welikson, and F. M. Quitkin, “Antidepressant Treatment of Primary Depression in Alcoholics: A Placebo-Controlled Randomized Clinical Trial,” *Psychopharmacology Bulletin*, 1995. pp. 598.

**McLean 1986**

***Main reference***

McLean, P. C., R. J. Ancill, and T. K. Szulecka, “Mianserin in the Treatment of Depressive Symptoms in Alcoholics: A Double-Blind Placebo Controlled Study Using a Computer Delivered Self-Rating Scale,” *Psychiatria Polska,* Vol. 20, No. 6, November–December 1986, pp. 417–427.

**Mielke 1978**

***Main reference***

Mielke, D. H., and D. M. Gallant, “Controlled Evaluation of a Pyrrolidine Derivative (AHR-1118) Versus Imipramine,” *Current Therapeutic Research—Clinical and Experimental,* Vol. 24, No. 6, 1978, pp. 734–737.

**Moak 2003**

***Main reference***

Moak, D. H., R. F. Anton, P. K. Latham, K. E. Voronin, R. L. Waid, and R. Durazo-Arvizu, “Sertraline and Cognitive Behavioral Therapy for Depressed Alcoholics: Results of a Placebo-Controlled Trial,” *Journal of Clinical Psychopharmacology,* Vol. 23, No. 6, December 2003, pp. 553–562.

***Additional references***

National Institute on Alcohol Abuse and Alcoholism, “Sertraline and Cognitive Therapy in Depressed Alcoholics,” 2005. As of March 19, 2019: <https://clinicaltrials.gov/show/NCT00000458>

**Muhonen 2008**

***Main reference***

Muhonen, L. H., J. Lonnqvist, K. Juva, and H. Alho, “Double-Blind, Randomized Comparison of Memantine and Escitalopram for the Treatment of Major Depressive Disorder Comorbid with Alcohol Dependence,” *Journal of Clinical Psychiatry,* Vol. 69, No. 3, 2008, pp. 392–399.

***Additional references***

Muhonen, L. H., J. Lahti, H. Alho, J. Lonnqvist, J. Haukka, and S. T. Saarikoski, “Serotonin Transporter Polymorphism as a Predictor for Escitalopram Treatment of Major Depressive Disorder Comorbid with Alcohol Dependence,” *Psychiatry Research,* Vol. 186, No. 1, March 30, 2011, pp. 53–57.

Muhonen, L. H., J. Lahti, D. Sinclair, J. Lonnqvist, and H. Alho, “Treatment of Alcohol Dependence in Patients with Co-Morbid Major Depressive Disorder—Predictors for the Outcomes with Memantine and Escitalopram Medication,” *Substance Abuse Treatment, Prevention, and Policy,* Vol. 3, October 3, 2008, p. 20.

Muhonen, L. H., J. Lonnqvist, J. Lahti, and H. Alho, “Age at Onset of First Depressive Episode as a Predictor for Escitalopram Treatment of Major Depression Comorbid with Alcohol Dependence,” *Psychiatry Research,* Vol. 167, No. 1–2, May 15, 2009, pp. 115–122.

NCT00368862, “Efficacy Study of Memantine Hydrochloride and Escitalopram for the Treatment of Co-Morbid Depression and Alcoholism,” 2006. As of March 19, 2019:
http://clinicaltrials.gov/show/NCT00368862

**O'Reilly 2019**

***Main reference***

O'Reilly, H., Hagerty, A., O'Donnell, S., Farrell, A., Hartnett, D., Murphy, E., Kehoe, E., Agyapong, V., McLoughlin, D. M., Farren, C.. "Alcohol Use Disorder and Comorbid Depression: A Randomized Controlled Trial Investigating the Effectiveness of Supportive Text Messages in Aiding Recovery," Alcohol Alcohol. 2019. 54:551-558.

***Additional references***

D. Hartnett, E. Murphy, E. Kehoe, V. Agyapong, D. M. McLoughlin, C. Farren. "Supportive text messages for patients with alcohol use disorder and a comorbid depression: a protocol for a single-blind randomised controlled aftercare trial," *BMJ Open*. 2017. 7:e013587.

Farren, C. K., O'Reilly, H., Hagerty, A., Agyapong, V., McLoughlin, D. "Supportive text messages for patientswith alcohol use disorder and comorbid depression. Six month randomised controlled trial with aftercare," *Alcoholism: Clinical and Experimental Research*. 2018. 42:112A.

NCT02404662, "Trial of Supportive Text Messages for Patients With Alcohol Use Disorder and a Co-morbid Depression," 2015. https://clinicaltrials.gov/ct2/show/record/NCT02404662

**Oslin 2005**

***Main reference***

Oslin, D. W., “Treatment of Late-Life Depression Complicated by Alcohol Dependence,” *American Journal of Geriatric Psychiatry,* Vol. 13, No. 6, June 2005, pp. 491–500.

***Additional references***

“Curbing Drinking Alleviates Depression in Dually Diagnosed Seniors: Study Examines Effectiveness of Naltrexone Plus SSRI,” *Brown University Geriatric Psychopharmacology Update,* Vol. 9, No. 8, 2005, pp. 1–5.

Gopalakrishnan, R., J. Ross, C. O’Brien, and D. Oslin, “Course of Late-Life Depression with Alcoholism Following Combination Therapy,” *Journal of Studies on Alcohol and Drugs,* Vol. 70, No. 2, March 2009, pp. 237–241.

NCT00018824, “Treating Alcohol Use in Older Adults with Depression,” 2001. As of March 19, 2019: http://clinicaltrials.gov/show/NCT00018824

**Petersen 2009**

***Main reference***

Petersen, C. L., and R. D. Zettle, “Treating Inpatients with Comorbid Depression and Alcohol Use Disorders: A Comparison of Acceptance and Commitment Therapy Versus Treatment as Usual,” *The Psychological Record,* Vol. 59, No. 4, Fall 2009, pp. 521–536.

***Additional references***

Petersen, C. L., *Treatment of Comorbid Depression and Alcohol Use Disorders in an Inpatient Setting: Comparison of Acceptance and Commitment Therapy Versus Treatment as Usual*, ProQuest Information & Learning, 2008.

**Pettinati 2010**

***Main reference***

Pettinati, H. M., D. W. Oslin, K. M. Kampman, W. D. Dundon, H. Xie, T. L. Gallis, C. A. Dackis, and C. P. O’Brien, “A Double-Blind, Placebo-Controlled Trial Combining Sertraline and Naltrexone for Treating Co-Occurring Depression and Alcohol Dependence,” *American Journal of Psychiatry,* Vol. 167, No. 6, June 2010, pp. 668–675.

***Additional references***

Dundon, W. D., H. M. Pettinati, and J. K. Garson, “An Examination of Potential Trends of Racial Response to Pharmacological Treatment for Depressed Alcoholics,” *Alcoholism: Clinical and Experimental Research,* Vol. 34, No. 6, 2010, p. 176A.

Plebani, J. G., H. M. Pettinati, K. M. Kampman, W. D. Dundon, and K. G. Lynch, “Relationship Between Gender, Relapse to Drinking and Subsequent Depression in a Pharmacotherapy Trial for Comorbid Depression and Alcohol Dependence,” *Alcoholism: Clinical and Experimental Research,* Vol. 32, No. 6, June 2008, pp. 193A.

Roth, T. L., E. M. Mahoney, W. D. Dundon, and H. M. Pettinati, “Combination Pharmacotherapy Sertraline and Naltrexone Decreases Suicidal Ideation Scores in Co-Morbid Depressed Alcoholics,” *Alcoholism: Clinical and Experimental Research,* Vol. 36, 2012, p. 245A.

“Sertraline Plus Naltrexone Superior for Depressed Alcohol-Dependent Patients,” *Brown University Psychopharmacology Update,* Vol. 21, No. 9, 2010, pp. 1–7.

University of Pennsylvania, “Sertraline for Alcohol Dependence and Depression,” January 2000. As of March 19, 2019: https://clinicaltrials.gov/show/NCT00004554

**Ralevski 2013**

***Main reference***

Ralevski, E., J. S. Jane, E. O’Brien, E. Edens, B. Arnaout, K. Kerfoot, K. Keegan, J. Weiner, M. Russo, and I. Petrakis, “Mecamylamine for Treatment of People with Dual Diagnoses of Depression and Alcohol Dependence,” *Journal of Dual Diagnosis,* Vol. 9, No. 4, 2013, pp. 301–310.

***Additional references***

Ralevski, E., J. S. Jane, A. O’Brien, J. Weiner, E. Edens, B. Arnaout, K. Kerfoot, R. Dwan, and I. Petrakis, “Mec1005mylamine for the treatment of patients with depression and alcohol dependence,” *Alcoholism: Clinical and Experimental Research,* Vol. 36, 2012, p. 296A.

Yale University and National Alliance for Research on Schizophrenia and Depression, “Mecamylamine for the Treatment of Patients with Depression and Alcohol Dependence,” August 2007. As of March 19, 2019:
https://clinicaltrials.gov/show/NCT00563797

**Roy 1998**

***Main reference***

Roy, A., “Placebo-Controlled Study of Sertraline in Depressed Recently Abstinent Alcoholics,” *Biological Psychiatry,* Vol. 44, No. 7, October 1, 1998, pp. 633–637.

**Roy-Byrne 2000**

***Main reference***

Roy-Byrne, P. P., K. P. Pages, J. E. Russo, C. Jaffe, A. W. Blume, E. Kingsley, D. S. Cowley, and R. K. Ries, “Nefazodone Treatment of Major Depression in Alcohol-Dependent Patients: A Double-Blind, Placebo-Controlled Trial,” *Journal of Clinical Psychopharmacology,* Vol. 20, No. 2, April 2000, pp. 129–136.

**Salloum 2007**

***Main reference***

Salloum, I. M., “Drug Treatment for Depressed Alcoholics (Naltrexone/Fluoxetine),” ClinicalTrials.gov identifier NCT00006204,” 2007. As of March 19, 2019:
https://clinicaltrials.gov/ct2/show/NCT00006204

***Additional references***

Salloum, I. M., J. R. Cornelius, A. Douaihy, R. Caceda, F. Miao, and L. Kirisci, “Differential Impact of Cannabis Abuse on Depression Remission in Comorbid Depression and Alcoholism,” *Alcoholism: Clinical and Experimental Research,* Vol. 36, 2012, p. 299A.

Salloum, I. M., J. R. Cornelius, A. Douaihy, and D. C. Daley, “Predicting Medication Adherence and Treatment Completion Among Depressed Alcoholics,” *Alcoholism: Clinical and Experimental Research,* Vol. 37, 2013, p. 198A.

Salloum, I. M., J. R. Cornelius, A. Douaihy, and L. Kirisci, “Gender Difference in Alcohol Treatment Response in Patients with Comorbid Alcoholism and Depression,” *Alcoholism: Clinical and Experimental Research,* Vol. 35, 2011, p. 19A.

Salloum, I. M., J. Perel, J. R. Cornelius, A. Douaihy, D. C. Daley, T. M. Kelly, and L. Kirisci, “Naltrexone Decreases Plasma Concentration of Fluoxetine/Norfluoxetine in Depressed Alcoholics: Results from a Double-Blind, Placebo-Controlled Study,” *Alcoholism: Clinical and Experimental Research,* Vol. 30, No. 9, September 2006, pp. 165A.

Salloum, I. M., J. M. Perel, J. R. Cornelius, A. Douaihy, L. Kirisci, J. C. Helsel, and M. E. Thase, “Therapeutic Drug Monitoring (TDM) and Treatment Response in Major Depression with Comorbid Alcoholism,” *Neuropsychopharmacology,* Vol. 35, 2010, pp. S314–S315.

**Shaw 1975**

***Main reference***

Shaw, J. A., P. Donley, D. W. Morgan, and J. A. Robinson, “Treatment of depression in alcoholics,” *American Journal of Psychiatry,* Vol. 132, No. 6, June 1975, pp. 641–644.

**Thapinta 2014**

***Main reference***

Thapinta, D., S. Skulphan, and P. Kittrattanapaiboon, “Brief Cognitive Behavioral Therapy for Depression Among Patients with Alcohol Dependence in Thailand,” *Issues in Mental Health Nursing,* Vol. 35, No. 9, September 2014, pp. 689–693.

**Thapinta 2017**

***Main reference***

Thapinta, D., S. Skulphan, V. Kitsumban, and C. Longchoopol, “Cognitive Behavior Therapy Self-Help Booklet to Decrease Depression and Alcohol Use Among People with Alcohol Dependence in Thailand,” *Issues in Mental Health Nursing*, June 14, 2017, pp. 1–7.

**Witte 2012**

***Main reference***

Witte, J., K. Bentley, A. E. Evins, A. J. Clain, L. Baer, P. Pedrelli, M. Fava, and D. Mischoulon, “A Randomized, Controlled, Pilot Study of Acamprosate Added to Escitalopram in Adults with Major Depressive Disorder and Alcohol Use Disorder,” *Journal of Clinical Psychopharmacology,* Vol. 32, No. 6, December 2012, pp. 787–796.

***Additional references***

Massachusetts General Hospital and National Alliance for Research on Schizophrenia and Depression, “Acamprosate Added to Escitalopram and Behavioral Treatment for Comorbid Depression and Alcoholism,” March 2007. As of March 19, 2019: https://clinicaltrials.gov/show/NCT00452543

**Zielinski 1979**

***Main reference***

Zielinski, J. J., “Behavioral Treatments of Depression with Alcoholics Receiving Pharmacological Aversion,” *Behavior Therapist,* Vol. 2, No. 5, 1979, pp. 25–27.

**eMethods 5**. Link to Open Science Framework page with RMarkdown code and HTML output

Code: <https://osf.io/8fmw2/>

Output: <https://osf.io/bwyq8/>

**eTable 1**. Summary of Studies Included in the Pharmacological Intervention Network

| **Study** | **Country** | | **N** | | | **Age** | | **Female** | | **Depression** | **Alcohol** | **Stage** | **Setting** | **Sites** | **Intervention 1** | **Intervention 2** | **Weeks** | **Co-Intervention** |
| --- | --- | --- | --- | --- | --- | --- | --- | --- | --- | --- | --- | --- | --- | --- | --- | --- | --- | --- |
| ***SSRI vs placebo*** |  | |  | | |  | |  | |  |  |  |  |  |  |  |  |  |
| Adamson 2015 | New Zealand | | 138 | | | 44 | | 59% | | Major Depressive Episode (DSM-IV) | Alcohol Dependence (DSM-IV) | Outpatient | SUD | 8 | Citalopram | Pharmacologic placebo | 12 | Pharmacotherapy, outpatient program |
| Cornelius 1997 | USA | | 51 | | | 35 | | 49% | | Major Depressive Disorder (DSM-III) | Alcohol Dependence (DSM-III) | Inpatient + Outpatient | Both | 1 | Fluoxetine | Pharmacologic placebo | 12 | Inpatient + outpatient program |
| Gual 2003 | Spain | | 83 | | | 47 | | 53% | | Major Depressive Disorder and/or Dysthymic Disorder (DSM-IV/ICD-10) | Alcohol Dependence (DSM-IV/ICD-10) | Outpatient | SUD | 1 | Sertraline | Pharmacologic placebo | 24 | Outpatient program |
| Kranzler 2006 | USA | | 345 | | | 43 | | 36% | | Major Depressive Disorder (DSM-IV) | Alcohol Dependence (DSM-IV) | NR | NR | 13 | Sertraline | Pharmacologic placebo | 10 | Outpatient program |
| Krupitsky 2013 | Russia | | 60 | | | 42 | | 22% | | Depression Episode (ICD-10) | Alcohol Dependence (ICD-10) | Inpatient | SUD | 1 | Escitalopram | Pharmacologic placebo | 13 | Outpatient program |
| Moak 2003 | USA | | 82 | | | 42 | | 39% | | Major Depressive Episode or Dysthymic Disorder (DSM-III) | Alcohol Abuse or Dependence (DSM-III) | NR | NR | 1 | Sertraline | Pharmacologic placebo | 12 | Psychotherapy |
| Pettinati 2010 | USA | | 170 | | | 43 | | 38% | | Major Depressive Disorder (DSM-IV) | Alcohol Dependence (DSM-IV) | Outpatient | SUD | 1 | Sertraline | Pharmacologic placebo | 14 | Psychotherapy |
| Roy 1998 | USA | | 36 | | | 41 | | 8% | | Major Depressive Episode (DSM-III) | Alcohol Dependence (DSM-III) | Inpatient + Outpatient | SUD | 1 | Sertraline | Pharmacologic placebo | 6 | Inpatient + outpatient program |
| ***SSRI vs opioid antagonist + SSRI*** | | | |  |  | |  | |  | |  |  |  |  |  |  |  |  |
| Pettinati 2010 | | USA | | 170 | 43 | | 38% | | Major Depressive Disorder (DSM-IV) | | Alcohol Dependence (DSM-IV) | Outpatient | SUD | 1 | Sertraline | Naltrexone + Sertraline | 14 | Psychotherapy |
| Salloum 2007 | | USA | | 106 | NR | | 46% | | Major Depressive Disorder (DSM-IV) | | Alcohol Dependence (DSM-IV) | NR | NR | 1 | Fluoxetine | Naltrexone + Fluoxetine | 26 | Psychotherapy |
| ***SSRI vs AAP + SSRI*** | |  | |  |  | |  | |  | |  |  |  |  |  |  |  |  |
| Han 2013 | | South Korea | | 35 | 40 | | 34% | | Major Depressive Disorder (DSM-IV) | | Alcohol Dependence (DSM-IV) | Inpatient | Both | 2 | Escitalopram | Aripiprazole + Escitalopram | 6 | Detoxification, inpatient program |
| ***SSRI vs NMDA antagonist*** | | | |  |  | |  | |  | |  |  |  |  |  |  |  |  |
| Muhonen 2008 | | Finland | | 80 | 48 | | 45% | | Major Depressive Disorder (DSM-IV) | | Alcohol Dependence (DSM-IV) | Outpatient | SUD | 3 | Escitalopram | Memantine | 26 | Outpatient program |
| ***SSRI vs opioid antagonist*** | | | |  |  | |  | |  | |  |  |  |  |  |  |  |  |
| Pettinati 2010 | | USA | | 170 | 43 | | 38% | | Major Depressive Disorder (DSM-IV) | | Alcohol Dependence (DSM-IV) | Outpatient | SUD | 1 | Sertraline | Naltrexone | 14 | Psychotherapy |
| ***SSRI vs TCA*** | |  | |  |  | |  | |  | |  |  |  |  |  |  |  |  |
| Cocchi 1997 | | Italy | | 122 | 42 | | 22% | | "Depression" | | "Alcoholic" | Inpatient | SUD | 1 | Paroxetine | Amitryptiline | 3-4 | Detoxification |
| ***TCA vs placebo*** | |  | |  |  | |  | |  | |  |  |  |  |  |  |  |  |
| Butterworth 1971 | | USA | | 40 | 23-60 | | 0% | | Depression (Lehmann Depression Rating Scale of 10+ or Clinical Impression) | | "Alcoholic" | Inpatient | SUD | 1 | Imipramine | Pharmacologic placebo | 3 | Detoxification, inpatient program |
| Mason 1996 | | USA | | 28 | 39 | | 14% | | Major Depressive Disorder (DSM-III) | | Alcohol Dependence (DSM-III) | Outpatient | SUD | 2 | Desipramine | Pharmacologic placebo | 26 | Psychotherapy, self-help group |
|  | |  | |  |  | |  | |  | |  |  |  |  |  |  |  |  |
| ***TCA vs placebo (cont)*** | |  | |  |  | |  | |  | |  |  |  |  |  |  |  |  |
| McGrath 1996 | | USA | | 69 | 37 | | 51% | | Major Depressive Disorder, Dysthymic Disorder, or Depressive Disorder not Otherwise Specified (DSM-III) | | Alcohol Abuse or Dependence (DSM-III) | Outpatient | Depression | 1 | Imipramine | Pharmacologic placebo | 12 | Outpatient program |
| ***TCA vs MRI*** | |  | |  |  | |  | |  | |  |  |  |  |  |  |  |  |
| Mielke 1978 | | USA | | 20 | 37 | | NR | | Major Depressive Disorder (DSM-II) | | "Alcoholism" | Inpatient | SUD | 1 | Imipramine | AHR-1118 | 4 | Inpatient program |
| ***TCA vs TCA*** | |  | |  |  | |  | |  | |  |  |  |  |  |  |  |  |
| Loo 1988 | | France | | 129 | 38 | | 14% | | Major Depressive Episode or Dysthymic Disorder (DSM-III) | | Alcohol Abuse or Dependence (DSM-III) | NR | NR | 7 | Tianeptine | Amitriptyline | 4-8 | Pharmacotherapy |
| ***TCA vs TeCA*** | |  | |  |  | |  | |  | |  |  |  |  |  |  |  |  |
| Altintoprak 2008 | | Turkey | | 44 | 4 | | 8% | | Major Depressive Disorder (DSM-IV) | | Alcohol Dependence (DSM-IV) | Inpatient | SUD | 1 | Amitriptyline | Mirtazapine | 8 | Detoxification |
| ***TeCA vs placebo*** | |  | |  |  | |  | |  | |  |  |  |  |  |  |  |  |
| Cornelius 2016 | | USA | | 14 | 41 | | 29% | | Major Depressive Disorder (DSM-IV) | | Alcohol Abuse or Dependence (DSM-IV) | Outpatient | Both | 1 | Mirtazapine | Pharmacologic placebo | 12 | Psychotherapy |
| McLean 1986 | | UK | | 35 | 37 | | 31% | | Depression (Hamilton Depression Rating Scale of 17+) | | "Alcohol Dependence" | Inpatient | SUD | 1 | Mianserin | Pharmacologic placebo | 4 | Inpatient program |
| ***Opioid antagonist vs placebo*** | | | |  |  | |  | |  | |  |  |  |  |  |  |  |  |
| Oslin 2005 | | USA | | 74 | 63 | | 20% | | Major Depressive Disorder (DSM-IV) | | Alcohol Dependence (DSM-IV) | NR | NR | 1 | Naltrexone | Pharmacologic placebo | 12 | Psychotherapy, pharmacotherapy |
| Pettinati 2010 | | USA | | 170 | 43 | | 38% | | Major Depressive Disorder (DSM-IV) | | Alcohol Dependence (DSM-IV) | Outpatient | SUD | 1 | Naltrexone | Pharmacologic placebo | 14 | Psychotherapy |
| ***Opioid antagonist vs opioid antagonist + SSRI*** | | | | |  | |  | |  | |  |  |  |  |  |  |  |  |
| Pettinati 2010 | | USA | | 170 | 43 | | 38% | | Major Depressive Disorder (DSM-IV) | | Alcohol Dependence (DSM-IV) | Outpatient | SUD | 1 | Naltrexone + Sertraline | Naltrexone | 14 | Psychotherapy |
| ***SARI vs placebo*** | |  | |  |  | |  | |  | |  |  |  |  |  |  |  |  |
| Hernandez-Avila 2004 | | USA | | 41 | 43 | | 51% | | Major Depressive Disorder (DSM-IV) | | Alcohol Dependence (DSM-IV) | Outpatient | Both | 1 | Nefazodone | Pharmacologic placebo | 10 | Psychotherapy |
| Roy-Byrne 2000 | | USA | | 64 | 40 | | 55% | | Major Depressive Disorder (DSM-III) | | Alcohol Dependence (DSM-III) | Outpatient | Both | 1 | Nefazodone | Pharmacologic placebo | 12 | Psychotherapy |
| ***Opioid antagonist + SSRI vs placebo*** | | | |  |  | |  | |  | |  |  |  |  |  |  |  |  |
| Pettinati 2010 | | USA | | 170 | 43 | | 38% | | Major Depressive Disorder (DSM-IV) | | Alcohol Dependence (DSM-IV) | Outpatient | SUD | 1 | Naltrexone + Sertraline | Pharmacologic placebo | 14 | Psychotherapy |
| ***AAP vs placebo*** | |  | |  |  | |  | |  | |  |  |  |  |  |  |  |  |
| Golik-Gruber 2003 | | Croatia | | 40 | NR | | 0% | | "Depression" | | "Alcohol Addiction" | Inpatient | SUD | 1 | Sulpride | Pharmacologic placebo | 3 | Outpatient program |
| ***Glutamatergic antagonist vs placebo*** | | | | |  | |  | |  | |  |  |  |  |  |  |  |  |
| Witte 2012 | | USA | | 23 | 46 | | 43% | | Major Depressive Disorder (DSM-IV) | | Alcohol Abuse or Dependence (DSM-IV) | NR | NR | 1 | Acamprosate | Pharmacologic placebo | 12 | Outpatient program |
| ***nAChRs vs placebo*** | |  | |  |  | |  | |  | |  |  |  |  |  |  |  |  |
| Ralevski 2013 | | USA | | 21 | 50 | | 29% | | Major Depressive Disorder (DSM-IV) | | Alcohol Dependence (DSM-IV) | NR | NR | 1 | Mecamylamine | Pharmacologic placebo | 12 | Outpatient program |
| ***NRI vs placebo*** | |  | |  |  | |  | |  | |  |  |  |  |  |  |  |  |
| Altamura 1990 | | NR | | 30 | 45 | | 20% | | Dysthymic Disorder (DSM-III) | | Alcohol Dependence (DSM-III) | NR | NR | 1 | Viloxazine | Pharmacologic placebo | 12 | Inpatient + outpatient program |
| ***TCA + sedative vs placebo*** | | | |  |  | |  | |  | |  |  |  |  |  |  |  |  |
| Shaw 1975 | | USA | | 30 | 32 | | 0% | | Depression (BDI, MMPI, Zung’s Scale) | | Alcoholism (National Council on Alcoholism) | Inpatient + Outpatient | SUD | 1 | Chlordiazepoxide + Imipramine | Pharmacologic placebo | 4 | Inpatient + outpatient program |

Abbreviations: AAP = atypical anti-psychotic, BDI = Beck’s Depression Intervention, DSM = Diagnostic and Statistical Manual of Mental Disorders, ICD = International Classification of Diseases, MMPI = Minnesota Multiphasic Personality Inventory, MRI = monoamine reuptake inhibitor, n = sample size, nAChRs = non-selective non-competitive antagonists of the nicotinic acetylcholine receptor, NMDA = N-methyl-D-aspartate, NR = not reported, NRI = norepinephrine reuptake inhibitor, SARI = serotonin antagonist and reuptake inhibitor, SSRI = selective serotonin reuptake inhibitor, SUD = substance use disorder, TCA = tricyclic antidepressant, TeCA = tetracyclic antidepressant, UK = United Kingdom, USA = United States of America.

**eTable 2**. Summary of Studies Included in the Psychological Intervention Network

| **Study** | **Country** | **N** | **Age** | **Female** | | **Depression** | **Alcohol** | **Stage** | | **Setting** | | **Sites** | | **Intervention 1** | | **Intervention 2** | | **Weeks** | | **Co-Intervention** | |
| --- | --- | --- | --- | --- | --- | --- | --- | --- | --- | --- | --- | --- | --- | --- | --- | --- | --- | --- | --- | --- | --- |
| ***CBT vs no additional treatment*** | | |  | |  |  |  |  | |  | |  | |  | |  | |  | |  | |
| Thapinta 2014 | Thailand | 80 | 45 | 18% | | Mild Depression or Moderate Depression  (9Q of 7-18) | "Alcohol Dependence" | Outpatient | | Both | | 5 | | Brief CBT | | No additional treatment | | 3 | | Psychotherapy, pharmacotherapy | |
| Thapinta 2017 | Thailand | 350 | 39 | 12% | | Mild Depression  (PHQ-9 of 5-8) | Alcohol Dependence (DSM-IV) | Outpatient | | Both | | 5 | | CBT Self Help Book | | No additional treatment | | 1 | | Psychotherapy | |
| ***CBT vs placebo*** |  |  |  |  | |  |  |  | |  | |  | |  | |  | |  | |  | |
| Petersen 2009 | USA | 30 | 38 | 50% | | Major Depressive Disorder (DSM-IV) | Alcohol Abuse or Dependence (DSM-IV) | Inpatient | | SUD | | 1 | | Acceptance and commitment therapy | | Psychological placebo | | 3-4 | | Inpatient program | |
| ***IPT vs no additional treatment*** | | | | | | | | | | | | | | | | | | | | | |
| Holzhauer 2017 | USA | 48 | 37 | 100% | | Major Depressive Disorder (DSM-IV) | Alcohol Dependence (DSM-IV) | Outpatient | | SUD | | 1 | | Interpersonal therapy | | No additional treatment | | 16 | | Outpatient program | |
| ***IPT vs SP*** |  |  |  |  | |  |  |  | |  | |  | |  | |  | |  | |  | |
| Markowitz 2008 | USA | 26 | 38 | 31% | | Dysthymic Disorder (DSM-IV) | Alcohol Abuse or Dependence (DSM-IV) | Outpatient | | Both | | 1 | | Interpersonal therapy for dysthymic disorder | | Brief supportive psychotherapy | | 16 | | Pharmacotherapy, self-help group | |
| ***Self-management support vs no additional treatment*** | | | | | | | | | | | | | | | | | | | | | |
| O'Reilly 2019 | Ireland | 95 | 48 | 46% | | Major Depressive Episode (DSM-IV) | Alcohol Dependence (DSM-IV) | Outpatient | | Both | | 1 | | Supportive text messaging | | No additional treatment | | 26 | | Outpatient aftercare program (support group) | |
| ***Self-management support vs placebo*** | | | | | |  |  | |  | |  | |  | |  | |  | |  | |  |
| Agyapong 2012 | Ireland | 54 | 49 | 54% | | Major Depressive Disorder (DSM-IV) | Alcohol Abuse or Dependence (DSM-IV) | Outpatient | | Both | | 1 | | Supportive text messaging | | Psychological placebo | | 13 | | Inpatient + outpatient program | |
| Zielinski 1979 | USA | 36 | 40 | 25% | | Depression (BDI, MMPI, Zung’s Self-Rating Depression Scale) | "Alcoholic" | Inpatient + Outpatient | | SUD | | 1 | | Activity Level Monitoring + Social Skills Training | | Psychological placebo | | 13 | | Inpatient + outpatient program | |
| Zielinski 1979 | USA | 36 | 40 | 25% | | Depression (BDI, MMPI, Zung’s Self-Rating Depression Scale) | "Alcoholic" | Inpatient + Outpatient | | SUD | | 1 | | Activity Level Monitoring | | Psychological placebo | | 13 | | Inpatient + outpatient program | |

Abbreviations: BDI = Beck’s Depression Inventory, CBT = cognitive behavioral therapy, DSM = Diagnostic and Statistical Manual of Mental Disorders, IPT = interpersonal therapy, MMPI = Minnesota Multiphasic Personality Inventory, n = sample size, PHQ = Patient Health Questionnaire, SP = supportive psychotherapy, SUD = substance use disorder, USA = United States of America.

**eTable 3**. Summary of Outcome Data Across Studies

| **Study** | **Remission from depression** | **Remission from alcohol use** | **Depressive symptoms** | **Total**  **drinking** | **Heavy**  **drinking** | **Withdrawal/craving**  **symptoms** | **Health-related quality of life** | **Functional**  **status** | **Adverse**  **events** |
| --- | --- | --- | --- | --- | --- | --- | --- | --- | --- |
| Adamson 2015 | Proportion with MADRS <10 at post-intervention | Mean percentage days abstinent at  post-intervention | Mean MADRS at post-intervention | Mean drinks per drinking day at  post-intervention | Mean percentage of days heavy drinking at post-intervention | Mean LDQ at post-intervention | - | - | Proportion self-reporting at least one adverse event at post-intervention |
| Agyapong 2012 | - | Proportion abstinent at immediately and 3-months post-intervention | Mean BDI at immediately and 3-months post-intervention | Mean units of alcohol per drinking day at immediately and 3-months post-intervention | - | Mean OCDS at immediately and 3-months post-intervention | - | Mean GAF at immediately and 3-months post-intervention | - |
| Altamura 1990 | - | - | - | - | - | - | - | - | - |
| Altintoprak 2008 | - | - | - | - | - | Mean ACS at post-intervention | - | - | - |
| Butterworth 1971 | - | - | Mean LRDR at post-intervention | - | - | - | - | Proportion with “improvement in global response” at post-intervention | Proportion with at least one physician-recorded adverse event at post-intervention |
| Cocchi  1997 | Proportion “not depressed” using ZDS at post-intervention | - | Mean ZDS at post-intervention | - | - | - | - | - | - |
| Cornelius 1997 | - | Proportion abstinent at  post-intervention  Mean number of days abstinent at 9-months post-intervention | Mean HDRS at immediately and 9-months post-intervention | Mean drinks per drinking day at  post-intervention  Number drinks in the past week at 9-months post-intervention | Cumulative number of days heavy drinking at post-intervention | - | - | Mean GAS at immediately and 9-months post-intervention | Proportion with at least one side effect at immediately and 9-months post-intervention |
| Cornelius 2016 | - | - | Mean BDI at post-intervention | Mean drinks per drinking day at  post-intervention | Mean number of days heavy drinking per week at post-intervention | Mean OCDS at post-intervention | - | - | - |
| Golik-Gruber 2003 | Proportion with depression (BDI) at post-intervention | - | - | - | - | - | - | - | - |
| Gual  2003 | Proportion with ≥50% reduction on MADRS at post-intervention | Proportion who did not relapse at post-intervention | - | - | - | - | - | - | - |
| Han  2013 | Proportion with ≥50% reduction on BDI at  post-intervention | Proportion who did not relapse at post-intervention | Mean BDI at post-intervention | - | - | Mean KAUQ at post-intervention | - | Mean CGI-severity at post-intervention | Proportion who dropped out due to adverse events at post-intervention |
| Hernandez-Avila 2004 | - | Proportion abstinent at  post-intervention | Mean HDRS at post-intervention | Mean drinks per drinking day at  post-intervention | Mean number of days heavy drinking per week at post-intervention | - | - | - | Mean SAFTEE score at post-intervention |
| Holzhauer 2017 | - | - | - | - | - | - | - | - | - |
| Kranzler  2006 | Proportion with ≥50% reduction on HDRS at post-intervention | Mean percentage days abstinent at  post-intervention | Mean HDRS at post-intervention | - | - | - | - | - | Proportion with a treatment emergent adverse event at post-intervention |
| Krupitsky 2013 | - | Mean number of days remission at  post-intervention | Mean HDRS at post-intervention | - | Mean GGT activity at post-intervention | Mean OCDS at post-intervention | - | Proportion with considerable or very considerable improvement on CGI at post-intervention | Proportion with at least one adverse event at post-intervention |
| Loo 1988 | Proportion with ≥50% reduction on MADRS at post-intervention | - | - | - | - | - | - | - | Proportion who discontinued treatment at post-intervention |
| Markowitz 2008 | Proportion with “remission” at post-intervention | Mean percentage days abstinent at  post-intervention | Mean HDRS at post-intervention | - | - | - | - | - | - |
| Mason  1996 | Proportion with “depression response” at post-intervention | Proportion who did not relapse at post-intervention | Mean HDRS at post-intervention | - | - | - | - | - | Proportion who dropped out due to adverse events at post-intervention |
| McGrath 1996 | Proportion with ≥50% reduction on HDRS at post-intervention | Proportion with past-week abstinence at  post-intervention | Mean HDRS at post-intervention | Mean drinks per drinking day at  post-intervention | Mean percentage of days heavy drinking in the past week at post-intervention | - | - | Proportion much improved or better on CGI at post-intervention | Proportion who dropped out due to adverse events at post-intervention |
| McLean 1986 | Proportion with HDRS <17 at post-intervention | - | Mean HDRS at post-intervention | - | - | - | - | - | Proportion who experienced transient drowsiness at post-intervention |
| Mielke  1978 | Proportion with moderate/marked improvement on the CGI at  post-intervention | - | - | - | - | - | - | - | Proportion with at least one side effect at post-intervention |
| Moak  2003 | Proportion with ≥50% reduction on BDI at  post-intervention | Mean percentage days abstinent at  post-intervention | Mean HDRS at post-intervention | Mean drinks per drinking day at  post-intervention | - | - | - | - | Proportion with at least one adverse event at post-intervention |
| Muhonen 2008 | Proportion with MADRS <12 at post-intervention | Mean AUDIT at  post-intervention | Mean MADRS at post-intervention | Mean daily grams alcohol at post-intervention | Mean number of days heavy drinking at post-intervention | Mean OCDS at post-intervention | Mean VAS at  post-intervention | Mean SOFAS at post-intervention | Proportion with at least one adverse event at post-intervention |
| O’Reilly 2019 | - | Proportion who have consumed any alcohol immediately and at 6-months post-intervention | BDI-II immediately and at 6-months post-intervention | Mean (1) days drinking in past 3 months and (2) units of alcohol per drinking day immediately and at 6-months post-intervention | - | Mean OCDS immediately and at 6-months post-intervention | - | - | - |
| Oslin  2005 | Proportion with HDRS <10 at post-intervention | Proportion abstinent at  post-intervention | - | - | Proportion relapsed to heavy drinking at post-intervention | - | - | - | - |
| Petersen 2009 | Proportion with HDRS <14 at post-intervention | - | Mean HDRS at post-intervention | - | - | - | - | - | - |
| Pettinati 2010 | Proportion “not depressed” at post-intervention | Proportion abstinent at  post-intervention | Mean HDRS at post-intervention | - | Mean days to relapse to heavy drinking at post-intervention | - | - | - | Proportion who discontinued treatment due to adverse events at post-intervention |
| Ralevski 2013 | - | - | Mean HDRS at post-intervention | - | - | - | - | - | Proportion who experienced at least one medical or psychiatric adverse event at post-intervention |
| Roy  1998 | Proportion with ≥50% reduction on HDRS at post-intervention | Proportion who did not relapse at post-intervention | Mean HDRS at post-intervention | - | - | - | - | Proportion very much improved on CGI at post-intervention | - |
| Roy-Byrne 2000 | Proportion with HDRS <8 at post-intervention | Proportion abstinent at  post-intervention | Mean HDRS at post-intervention | Mean drinks per drinking day at  post-intervention | - | Mean VAS at post-intervention | - | Proportion very much improved or much better on CGI at post-intervention | Mean number of adverse events per participant at post-intervention |
| Salloum 2007 | - | - | - | - | - | - | - | - | - |
| Shaw  1975 | - | - | - | - | - | - | - | - | - |
| Thapinta 2014 | - | - | Mean 9Q at immediately and 1-month post-intervention | - | - | - | - | - | - |
| Thapinta 2017 | - | - | Mean PHQ-9 at immediately,  1-month, and  6-month post-intervention | Mean cubic cm of alcohol per day at immediately, 3-months, and 6-months post-intervention | - | - | - | - | - |
| Witte  2012 | Proportion with “remission” at post-intervention | Mean percentage days abstinent at  post-intervention | Mean HDRS at post-intervention | Mean drinks per drinking day at  post-intervention | - | Mean OCDS at post-intervention | Mean Q-LESQ at post-intervention | Mean CGI-improvement at post-intervention | Proportion who experienced at least one adverse event at post-intervention |
| Zielinski 1979 | - | Proportion who did not relapse at 6- and 12-months post-intervention | - | - | - | - | - | - | - |

Abbreviations: ACS = Alcohol Craving Scale, AUDIT = Alcohol Use Disorder Identification Test, BDI = Beck Depression Inventory, CGI = Clinical Global Impression scale, cm = centimeters, GAF = Global Assessment of Function, GAS = Global Assessment Scale, GGT = Gamma-Glutamiltransferase, HDRS = Hamilton Depression Rating Scale, KAUQ = Korean Alcohol Urge Questionnaire, LDQ = Leeds Dependence Questionnaire, LRDR = Lehmann-Rockliff Depression Rating, MADRS = Montgomery-Åsberg Depression Rating Scale, OCDS = Obsessive Compulsive Drinking Scale, PHQ = Patient Health Questionnaire, Q-LESQ = Quality of Life Enjoyment and Satisfaction Questionnaire, SAFTEE = Systematic Assessment for Treatment of Emergent Events, SOFAS = Social and Occupational Functioning Assessment Scale, VAS = Visual Analog Scale, ZDS = Zung Depression Scale.

**eTable 4.** Summary of Risks of Bias Across Studies

| **Study** | **Random sequence generation** | **Allocation concealment** | **Blinding participants** | **Blinding providers** | **Blinding assessors** | **Completeness of**  **outcome data** | **Selective outcome reporting** | **Funder** |
| --- | --- | --- | --- | --- | --- | --- | --- | --- |
| Adamson 2015 | Low | Low | Low | Low | Low | Low | Low | Public |
| Agyapong 2012 | Low | Unclear | High | Low | High | Low | Low | Public |
| Altamura 1990 | Unclear | Unclear | Low | Low | Unclear | Low | High* | Unclear |
| Altintoprak 2008 | Unclear | Unclear | Low | Low | Unclear | Unclear | High* | Unclear |
| Butterworth 1971 | Unclear | Unclear | Low | Low | Unclear | Low | Unclear | Unclear |
| Cocchi 1997 | Unclear | Unclear | Unclear | Unclear | Unclear | Low | Unclear | Unclear |
| Cornelius 1997 | Unclear | Low | Low | Low | Low | Low | Unclear | Public |
| Cornelius 2016 | Unclear | Low | Low | Low | Unclear | Low | Low | Public |
| Golik-Gruber 2003 | Unclear | Unclear | High | High | High | Low | Unclear | Unclear |
| Gual 2003 | Unclear | Unclear | Low | Low | Unclear | High | High* | Unclear |
| Han 2013 | Unclear | Unclear | Low | Low | Unclear | High* | Unclear | Some private |
| Hernandez-Avila 2004 | Low | Unclear | High | Low | Low | Low | Unclear | Some private |
| Holzhauer 2017 | Unclear | Unclear | High | High | Unclear | Low | High* | Public |
| Kranzler 2006 | Low | Unclear | Low | Low | Unclear | High | High* | Some private |
| Krupitsky 2013 | Low | Low | Low | Low | Low | Low | High* | Unclear |
| Loo 1988 | Unclear | Unclear | Low | Low | Unclear | Low | Unclear | Unclear |
| Markowitz 2008 | Low | Unclear | High | High | Low | High | Unclear | Public |
| Mason 1996 | Unclear | Unclear | Low | Low | Unclear | High | High* | Public |
| McGrath 1996 | Unclear | Unclear | Low | Low | Low | High | Unclear | Some private |
| McLean 1986 | Unclear | Unclear | Low | Low | Low | Low | Unclear | Unclear |
| Mielke 1978 | Unclear | Unclear | Low | Low | Unclear | Low | High* | Public |
| Moak 2003 | Unclear | Unclear | Low | Low | Unclear | Unclear | High* | Some private |
| Muhonen 2008 | Low | Low | Low | Low | Unclear | High | Low | Some private |
| O’Reilly 2019 | Low | Low | High | High | Low | High | High* | Public |
| Oslin 2005 | Unclear | Unclear | Unclear | Unclear | Unclear | High | High* | Some private |
| Petersen 2009 | Low | Low | High | High | Low | High | High* | Unclear |
| Pettinati 2010 | Unclear | Unclear | Low | Low | Unclear | High* | High* | Some private |
| Ralevski 2013 | Unclear | Unclear | Low | Low | Low | Low | High* | Public |
| Roy 1998 | Unclear | Unclear | Low | Low | Low | High* | High* | Unclear |
| Roy-Byrne 2000 | Unclear | Unclear | Low | Low | Low | High | High* | Some private |
| Salloum 2007 | Unclear | Unclear | Low | Low | Unclear | Unclear | High* | Public |
| Shaw 1975 | Unclear | Unclear | Low | Low | Unclear | Low | High* | Unclear |
| Thapinta 2014 | Unclear | Unclear | High | High | Unclear | High | Unclear | Public |
| Thapinta 2017 | Unclear | Unclear | High | High | Low | Low | Unclear | Public |
| Witte 2012 | Unclear | Unclear | Low | Low | Unclear | High | Low | Some private |
| Zielinski 1979 | Unclear | Unclear | High | High | Unclear | Low | Unclear | Unclear |

* indicates that risk of bias is high for only some outcomes/time-points.

**eTable 5.** Pharmacological Intervention Effect Estimates at Post-Intervention for Remission from Depression and Alcohol Use

| **AAP** | 0.95  [0.0518; 17.3812] | 1.37  [0.0730; 25.7568] | 1.58  [0.0630; 39.4245] | 1.58  [0.1050; 23.7934] | 1.13  [0.1162; 11.0487] | 0.37  [0.0293; 4.7676] | 1.71  [0.2352; 12.4941] | 0.36  [0.0239; 5.3310] | 1.29  [0.1629; 10.2722] | 0.68  [0.0695; 6.5611] | 1.32  [0.0814; 21.4972] |
| --- | --- | --- | --- | --- | --- | --- | --- | --- | --- | --- | --- |
| - | **AAP + SSRI** | 1.45  [0.0700; 29.8662] | 1.66  [0.0638; 43.2700] | 1.67  [0.1134; 24.4897] | 1.19  [0.1130; 12.6279] | 0.39  [0.0293; 5.2945] | 1.81  [0.2160; 15.1217] | 0.38  [0.0228; 6.2299] | 1.36  [0.1772; 10.4937] | 0.71  [0.0693; 7.3201] | 1.39  [0.0776; 25.0460] |
| - | 0.27  [0.0233; 3.2247] | **Glutamatergic antagonist** | 1.15  [0.0412; 32.0451] | 1.15  [0.0674; 19.7206] | 0.83  [0.0729; 9.3706] | 0.27  [0.0186; 3.9827] | 1.25  [0.1445; 10.8158] | 0.26  [0.0153; 4.4201] | 0.94  [0.1007; 8.8340] | 0.49  [0.0436; 5.5659] | 0.96  [0.0524; 17.7572] |
| - | - | - | **MRI** | 1.00  [0.0459; 21.9430] | 0.72  [0.0459; 11.2739] | 0.24  [0.0121; 4.6248] | 1.09  [0.0863; 13.7055] | 0.23  [0.0099; 5.1707] | 0.82  [0.0646; 10.4263] | 0.43  [0.0439; 4.1878] | 0.84  [0.0342; 20.6169] |
| - | 0.98  [0.1194; 7.9900] | 3.56  [0.5921; 21.4583] | - | **NMDA antagonist** | 0.72  [0.0869; 5.9154] | 0.24  [0.0219; 2.5435] | 1.08  [0.1712; 6.8669] | 0.23  [0.0168; 3.0466] | 0.82  [0.1424; 4.7024] | 0.43  [0.0534; 3.4177] | 0.84  [0.0568; 12.3250] |
| - | 0.41  [0.0537; 3.1103] | 1.49  [0.2758; 8.0635] | - | 0.42**  [0.1359; 1.2876] | **Opioid antagonist** | 0.33  [0.0605; 1.7961] | 1.51  [0.4967; 4.6073] | 0.32  [0.0369; 2.6927] | 1.14  [0.3502; 3.7198] | 0.60  [0.1275; 2.7870] | 1.17  [0.1228; 11.0914] |
| - | 1.67  [0.2082; 13.4557] | ****6.11***  ***[1.0532; 35.4318]*** | - | 1.71  [0.5060; 5.8048] | ****4.10***  ***[1.6431; 10.2123]*** | **Opioid antagonist + SSRI** | 4.59 **  [0.9324; 22.5961] | 0.96  [0.0842; 10.8561] | 3.46**  [0.6929; 17.3095] | 1.81  [0.2689; 12.1625] | 3.54  [0.2838; 44.1849] |
| - | 0.57  [0.0842; 3.9245] | 2.10  [0.4473; 9.8391] | - | 0.59**  [0.2361; 1.4669] | 1.41  [0.7139; 2.7719] | ****0.34***  ***[0.1486; 0.7937]*** | **Pharmacological placebo** | 0.21  [0.0333; 1.3033] | 0.75  [0.4181; 1.3616] | 0.39**  [0.1303; 1.1911] | 0.77  [0.1090; 5.4606] |
| - | 0.76  [0.0862; 6.6570] | 2.76  [0.4348; 17.5799] | - | 0.78  [0.1978; 3.0418] | 1.85  [0.5463; 6.2925] | 0.45  [0.1212; 1.6895] | 1.32  [0.4769; 3.6421] | **SARI** | 3.62  [0.5277; 24.8570] | 1.89  [0.2222; 16.0969] | 3.70  [0.2534; 54.1027] |
| - | 0.54  [0.0804; 3.6471] | 1.98  [0.4143; 9.4316] | - | 0.55  [0.2292; 1.3420] | 1.33  [0.6616; 2.6561] | ****0.32***  ***[0.1395; 0.7504]*** | ***0.94*****  ***[0.7482; 1.1868]*** | 0.71  [0.2521; 2.0275] | **SSRI** | 0.52  [0.1695; 1.6084] | 1.02  [0.1324; 7.8959] |
| - | 1.02  [0.1121; 9.3726] | 3.74  [0.5613; 24.9210] | - | 1.05  [0.2513; 4.3813] | 2.51  [0.6892; 9.1274] | 0.61  [0.1537; 2.4390] | 1.78  [0.5938; 5.3527] | 1.35  [0.3026; 6.0461] | 1.89  [0.6152; 5.8179] | **TCA** | 1.96  [0.2068; 18.5403] |
| - | - | - | - | - | - | - | - | - | - | - | **TeCA** |

**Notes**: The upper right triangle provides estimates for remission from depression, and the bottom left triangle provides estimates for remission from alcohol use. OR < 1 favors the intervention in the column, and OR > 1 favors the intervention in the row. *******indicates that the confidence interval does not include an estimate of no effect. ** indicates at least one sensitivity analysis with a substantively different estimate.

**eTable 6.** Pharmacological Intervention Effect Estimates at Post-Intervention for Depressive and Withdrawal/Craving Symptoms

| **AAP + SSRI** | -0.11  [-1.36; 1.13] | 0.41  [-0.86; 1.68] | 0.25  [-0.81; 1.31] | -0.28  [-1.29; 0.74] | -0.45  [-1.47; 0.57] | 0.21  [-0.63; 1.06] | -0.10  [-1.07; 0.88] | 0.07  [-0.74; 0.89] | -0.16  [-1.05; 0.74] | 0.09  [-0.98; 1.16] |
| --- | --- | --- | --- | --- | --- | --- | --- | --- | --- | --- |
| -0.55  [-1.67; 0.57] | **Glutamatergic antagonist** | 0.53  [-0.79; 1.85] | 0.36  [-0.80; 1.52] | -0.16  [-1.27; 0.94] | -0.34  [-1.44; 0.77] | 0.33  [-0.59; 1.25] | 0.02  [-1.03; 1.06] | 0.19  [-0.76; 1.13] | -0.04  [-1.03; 0.94] | 0.21  [-0.92; 1.33] |
| - | - | **nAChRs** | -0.17  [-1.35; 1.02] | -0.69  [-1.82; 0.44] | -0.86  [-2.00; 0.26] | -0.20  [-1.15; 0.75] | -0.51  [-1.58; 0.56] | -0.34  [-1.31; 0.63] | -0.57  [-1.58; 0.44] | -0.32  [-1.48; 0.83] |
| -0.22  [-1.06; 0.62] | 0.33  [-0.64; 1.30] | - | **NMDA antagonist** | -0.52  [-1.43; 0.38] | -0.70  [-1.60; 0.21] | -0.03  [-0.74; 0.67] | -0.34  [-1.20; 0.52] | -0.17  [-0.85; 0.50] | -0.40  [-1.17; 0.36] | -0.16  [-1.12; 0.81] |
| - | - | - | - | **Opioid antagonist** | -0.17  [-0.85; 0.50] | 0.49  [-0.12; 1.10] | 0.18  [-0.60; 0.97] | 0.35  [-0.25; 0.96] | 0.12  [-0.57; 0.81] | 0.37  [-0.52; 1.26] |
| - | - | - | - | - | **Opioid antagonist + SSRI** | ****0.66***  ***[ 0.06; 1.27]*** | 0.36**  [-0.43; 1.14] | 0.52  [-0.08; 1.13] | 0.30  [-0.39; 0.99] | 0.54  [-0.35; 1.44] |
| -0.57  [-1.34; 0.20] | -0.02  [-0.84; 0.80] | - | -0.35  [-0.88; 0.17] | - | - | **Pharmacological placebo** | -0.31  [-0.80; 0.19] | -0.14  [-0.35; 0.07] | ***-0.37*****  ***[-0.72; -0.02]*** | -0.12  [-0.78; 0.53] |
| -0.20  [-1.14; 0.73] | 0.35  [-0.63; 1.32] | - | 0.01  [-0.73; 0.76] | - | - | 0.37  [-0.16; 0.90] | **SARI** | 0.17  [-0.37; 0.71] | -0.06  [-0.67; 0.55] | 0.19  [-0.64; 1.01] |
| -0.49  [-1.21; 0.23] | 0.06  [-0.81; 0.92] | - | -0.27  [-0.72; 0.17] | - | - | 0.08  [-0.20; 0.36] | -0.29  [-0.89; 0.31] | **SSRI** | -0.23  [-0.59; 0.14] | 0.02  [-0.67; 0.70] |
| -1.05  [-2.51; 0.42] | -0.50  [-1.99; 0.99] | - | -0.83  [-2.18; 0.52] | - | - | -0.48  [-1.72; 0.77] | -0.84  [-2.20; 0.51] | -0.55  [-1.83; 0.72] | **TCA** | 0.25  [-0.50; 0.99] |
| -0.98  [-2.29; 0.33] | -0.43  [-1.77; 0.90] | - | -0.77  [-1.95; 0.41] | - | - | -0.41  [-1.47; 0.65] | -0.78  [-1.97; 0.40] | -0.49  [-1.59; 0.60] | 0.06  [-0.59; 0.72] | **TeCA** |

**Notes**: The upper right triangle provides estimates for depressive symptoms, and the bottom left triangle provides estimates for withdrawal/craving symptoms. SMD < 0 favors the intervention in the column, and SMD > 0 favors the intervention in the row. *******indicates that the confidence interval does not include an estimate of no effect. ** indicates at least one sensitivity analysis with a substantively different estimate.

**eTable 7.** Pharmacological Intervention Effect Estimates at Post-Intervention for Total and Heavy Drinking

| **Glutamatergic antagonist** | - | - | - | - | - | - | - | - |
| --- | --- | --- | --- | --- | --- | --- | --- | --- |
| ****2.23***  ***[1.16; 3.30]*** | **NMDA antagonist** | 0.49  [-0.26; 1.25] | -0.02  [-0.83; 0.79] | 0.58  [-0.09; 1.25] | -0.45  [-1.47; 0.57] | 0.46  [-0.14; 1.06] | 0.80  [-0.14; 1.74] | 0.97  [-0.35; 2.29] |
| - | - | **Opioid antagonist** | -0.51**  [-1.07; 0.04] | 0.09  [-0.33; 0.50] | ****-0.95***  ***[-1.82; -0.08]*** | -0.04  [-0.49; 0.41] | 0.31  [-0.48; 1.09] | 0.48  [-0.73; 1.68] |
| - | - | - | **Opioid antagonist + SSRI** | ****0.60***  ***[ 0.08; 1.13]*** | -0.43  [-1.37; 0.50] | 0.48  [-0.06; 1.02] | 0.82**  [-0.03; 1.67] | 0.99  [-0.26; 2.24] |
| -0.00  [-0.85; 0.85] | ***-2.23*****  ***[-2.87; -1.58]*** | - | - | **Pharmacological placebo** | ****-1.04***  ***[-1.80; -0.27]*** | -0.13  [-0.43; 0.17] | 0.22  [-0.45; 0.88] | 0.39  [-0.74; 1.52] |
| 0.23  [-0.73; 1.18] | ***-2.00*****  ***[-2.78; -1.22]*** | - | - | 0.23  [-0.21; 0.66] | **SARI** | ****0.91***  ***[ 0.09; 1.73]*** | ****1.25***  ***[ 0.24; 2.27]*** | ****1.43***  ***[ 0.06; 2.79]*** |
| 0.30  [-0.59; 1.20] | ***-1.92*****  ***[-2.51; -1.34]*** | - | - | ****0.30***  ***[ 0.02; 0.59]*** | 0.08  [-0.44; 0.60] | **SSRI** | 0.34  [-0.39; 1.07] | 0.51  [-0.66; 1.69] |
| 0.09  [-0.94; 1.12] | ***-2.14*****  ***[-3.01; -1.27]*** | - | - | 0.09  [-0.49; 0.66] | -0.14  [-0.86; 0.58] | -0.22  [-0.86; 0.43] | **TCA** | 0.17  [-1.14; 1.48] |
| 0.54  [-0.84; 1.93] | ***-1.69*****  ***[-2.95; -0.42]*** | - | - | 0.54  [-0.55; 1.64] | 0.32  [-0.86; 1.49] | 0.24  [-0.89; 1.37] | 0.45  [-0.78; 1.69] | **TeCA** |

**Notes**: The upper right triangle provides estimates for heavy drinking, and the bottom left triangle provides estimates for alcohol use. SMD < 0 favors the intervention in the column, and SMD > 0 favors the intervention in the row. *******indicates that the confidence interval does not include an estimate of no effect. ** indicates at least one sensitivity analysis with a substantively different estimate.

**eTable 8.** Pharmacological Intervention Effect Estimates at Post-Intervention for Health-Related Quality of Life and Functional Status

| **AAP + SSRI** | - | - | - | - | - | - |
| --- | --- | --- | --- | --- | --- | --- |
| -0.95  [-2.12; 0.22] | **Glutamatergic antagonist** | - | 0.33  [-0.50; 1.1504] | - | - | - |
| 0.20  [-0.64; 1.03] | ***1.14*****  ***[ 0.11; 2.17]*** | **NMDA antagonist** | - | - | -0.09  [-0.53; 0.35] | - |
| ***-1.02*****  ***[-1.86; -0.18]*** | -0.07  [-0.89; 0.75] | ****-1.21***  ***[-1.84; -0.59]*** | **Pharmacological placebo** | - | - | - |
| -0.39  [-1.42; 0.64] | 0.56  [-0.46; 1.58] | -0.58  [-1.45; 0.29] | ***0.63*****  ***[ 0.03; 1.24]*** | **SARI** | - | - |
| -0.10  [-0.81; 0.60] | 0.85  [-0.09; 1.78] | -0.30  [-0.74; 0.14] | ****0.92***  ***[ 0.47; 1.36]*** | 0.28  [-0.47; 1.04] | **SSRI** | - |
| -0.19  [-1.19; 0.80] | 0.76  [-0.22; 1.73] | -0.39  [-1.21; 0.44] | ***0.83*****  ***[ 0.29; 1.36]*** | 0.19  [-0.62; 1.00] | -0.09  [-0.79; 0.61] | **TCA** |

**Notes**: The upper right triangle provides estimates for health-related quality of life, and the bottom left triangle provides estimates for functional status. SMD < 0 favors the intervention in the column, and SMD > 0 favors the intervention in the row. *******indicates that the confidence interval does not include an estimate of no effect.

**eTable 9.** Pharmacological Intervention Effect Estimates at Post-Intervention for Adverse Events

| **AAP + SSRI** | 0.30  [0.01; 11.50] | 0.05  [0.001; 2.86] | 0.07  [0.001; 2.89] | 0.06  [0.001; 2.94] | 0.13  [0.004; 4.22] | 0.60  [0.02; 16.26] | 0.12  [0.01; 2.27] | 0.16**  [0.01; 3.74] | 0.27  [0.02; 4.38] | 0.29  [0.01; 7.01] | 0.43  [0.01; 23.97] |
| --- | --- | --- | --- | --- | --- | --- | --- | --- | --- | --- | --- |
| - | **Glutamatergic antagonist** | 0.18  [0.01; 5.98] | 0.23  [0.01; 5.81] | 0.21  [0.01; 7.44] | 0.43  [0.02; 9.68] | 2.00  [0.11; 36.39] | 0.42  [0.05; 3.81] | 0.53  [0.04; 6.77] | 0.92  [0.09; 9.82] | 0.98  [0.07; 12.72] | 1.44  [0.04; 50.36] |
| - | - | **MRI** | 1.31  [0.04; 46.89] | 1.18  [0.02; 58.07] | 2.37  [0.07; 78.77] | 11.16  [0.41; 303.87] | 2.32  [0.15; 35.31] | 2.95  [0.15; 59.27] | 5.11  [0.30; 88.43] | 5.44  [0.50; 59.38] | 8.05  [0.16; 393.28] |
| - | - | - | **nAChRs** | 0.90  [0.02; 34.10] | 1.82  [0.07; 44.77] | 8.53  [0.43; 169.34] | 1.78  [0.17; 18.21] | 2.26  [0.16; 31.86] | 3.91  [0.33; 46.55] | 4.16  [0.29; 59.86] | 6.15  [0.16; 230.77] |
| - | - | - | - | **NMDA antagonist** | 2.01  [0.07; 60.89] | 9.46  [0.38; 233.56] | 1.97  [0.12; 32.10] | 2.50  [0.12; 53.54] | 4.33  [0.30; 61.74] | 4.62  [0.21; 100.33] | 6.82  [0.13; 350.23] |
| - | - | - | - | 3.43  [0.26; 45.48] | **Opioid antagonist** | 4.70  [0.54; 41.12] | 0.98  [0.11; 8.88] | 1.24  [0.10; 15.77] | 2.15  [0.25; 18.22] | 2.29  [0.18; 29.66] | 3.39  [0.10; 117.67] |
| - | - | - | - | 9.18  [0.63; 132.90] | 2.67  [0.86; 8.26] | **Opioid antagonist + SSRI** | 0.21**  [0.03; 1.36] | 0.26**  [0.03; 2.54] | 0.46  [0.08; 2.75] | 0.49  [0.05; 4.78] | 0.72  [0.03; 20.61] |
| - | - | - | - | 3.18  [0.25; 39.80] | 0.93  [0.39; 2.20] | 0.35  [0.12; 1.05] | **Pharmacological placebo** | 1.27  [0.36; 4.49] | 2.20**  [0.94; 5.16] | 2.34  [0.64; 8.61] | 3.46  [0.21; 55.75] |
| - | - | - | - | - | - | - | - | **SARI** | 1.73**  [0.38; 7.93] | 1.84**  [0.30; 11.30] | 2.72  [0.13; 57.67] |
| - | - | - | - | 2.05  [0.18; 23.59] | 0.60  [0.26; 1.39] | ****0.22***  ***[0.08; 0.66]*** | 0.64  [0.34; 1.23] | - | **SSRI** | 1.07  [0.22; 5.05] | 1.57  [0.09; 28.82] |
| - | - | - | - | 3.18  [0.03; 351.16] | 0.93  [0.02; 53.77] | 0.35  [0.01; 21.31] | 1.00  [0.02; 52.85] | - | 1.55  [0.03; 86.37] | **TCA** | 1.48  [0.07; 31.80] |
| - | - | - | - | 3.18  [0.03; 376.08] | 0.93  [0.01; 58.20] | 0.35  [0.01; 23.04] | 1.00  [0.02; 57.31] | - | 1.55  [0.03; 93.57] | 1.00  [0.004; 289.57] | **TeCA** |

**Notes**: The upper right triangle provides estimates for adverse events, and the bottom left triangle provides estimates for serious adverse events. OR < 1 favors the intervention in the column, and OR > 1 favors the intervention in the row. *******indicates that the confidence interval does not include an estimate of no effect.

**eFigure 1**. Forest Plot of Effect Estimates (Active Pharmacological Interventions versus Pharmacological Placebo) on Remission from Depression at Post-Intervention


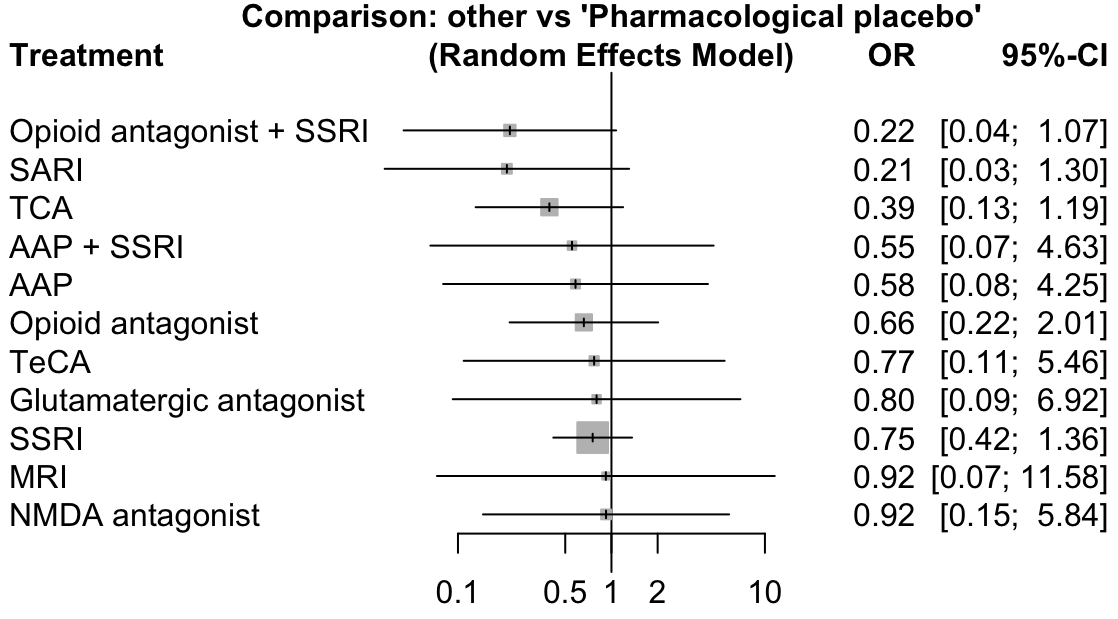


**eFigure 2.** Forest Plot of Effect Estimates (Active Pharmacological Interventions versus Pharmacological Placebo) on Remission from Alcohol Use at Post-Intervention


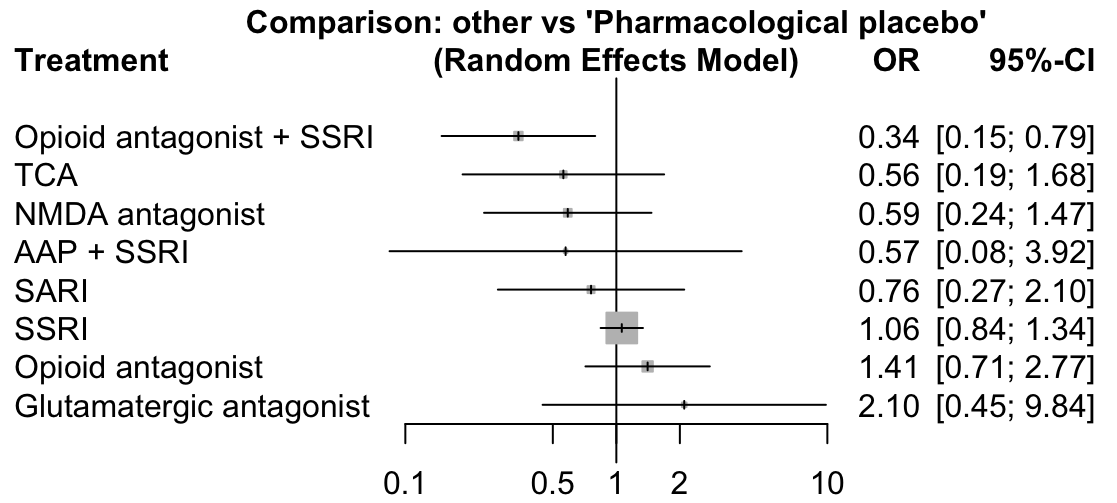


**eFigure 3.** Forest Plot of Effect Estimates (Active Pharmacological Interventions versus Pharmacological Placebo) on Depressive Symptoms at Post-Intervention


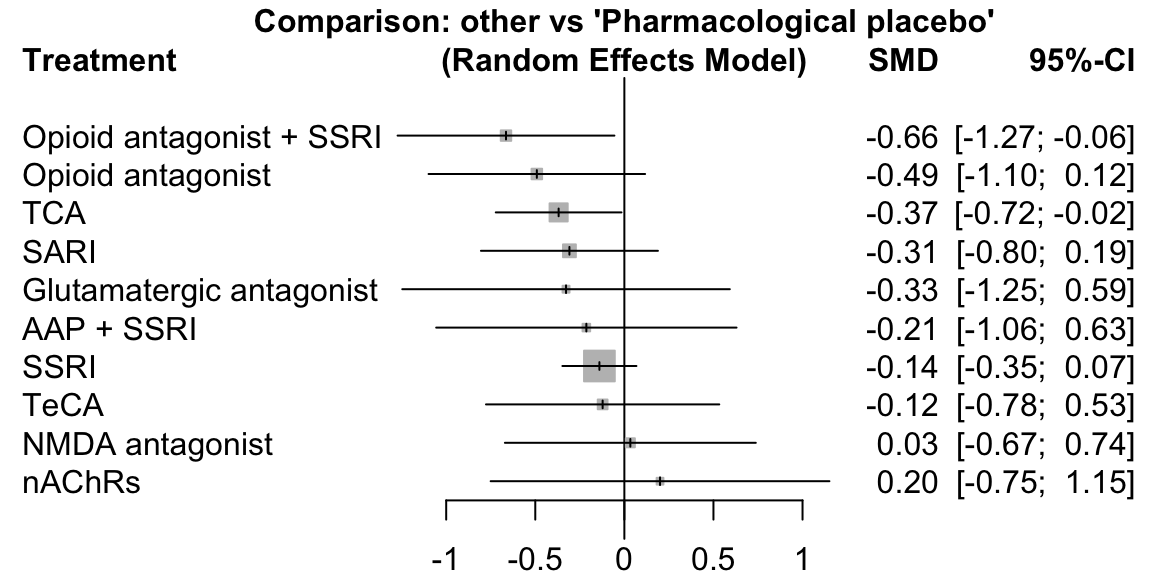


**eFigure 4.** Forest Plot of Effect Estimates (Active Pharmacological Interventions versus Pharmacological Placebo) on Withdrawal/Craving Symptoms at Post-Intervention


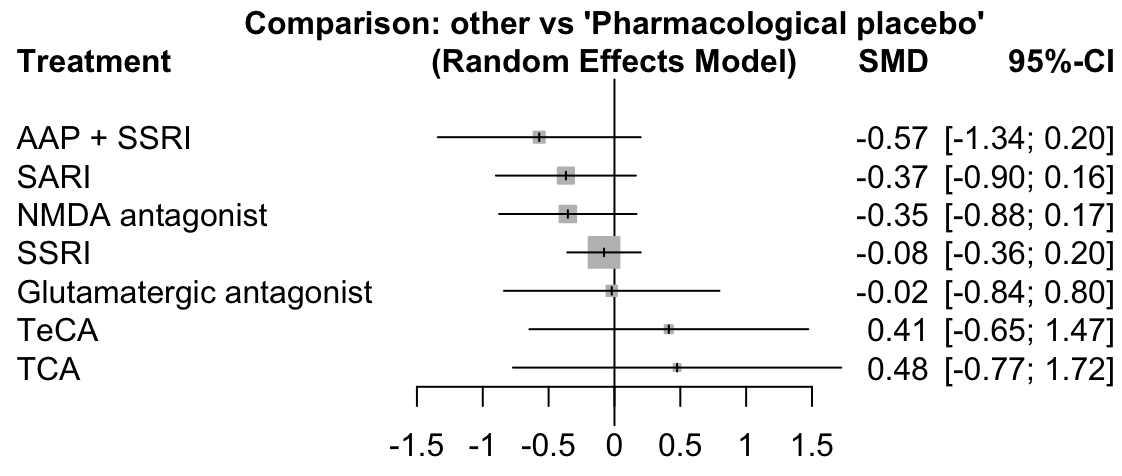


**eFigure 5.** Forest Plot of Effect Estimates (Active Pharmacological Interventions versus Pharmacological Placebo) on Alcohol Use at Post-Intervention


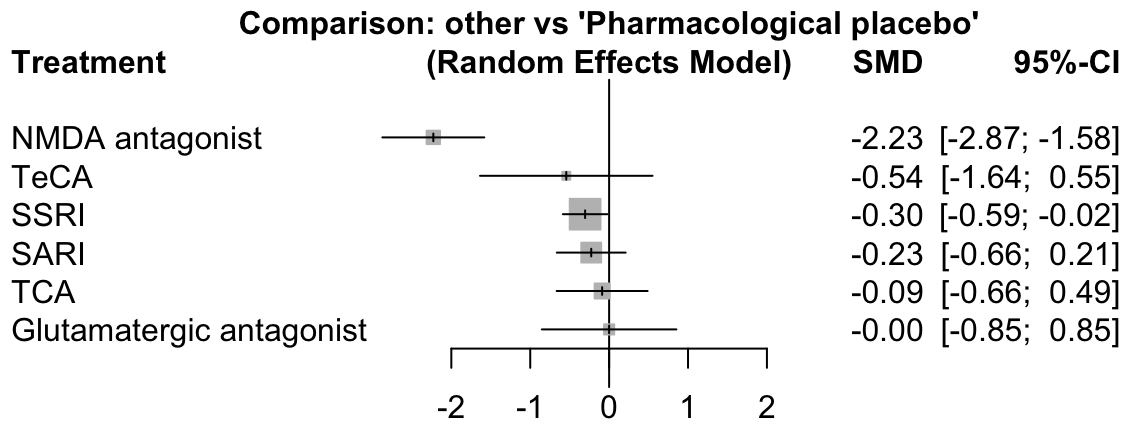


**eFigure 6.** Forest Plot of Effect Estimates (Active Pharmacological Interventions versus Pharmacological Placebo) on Heavy Drinking at Post-Intervention


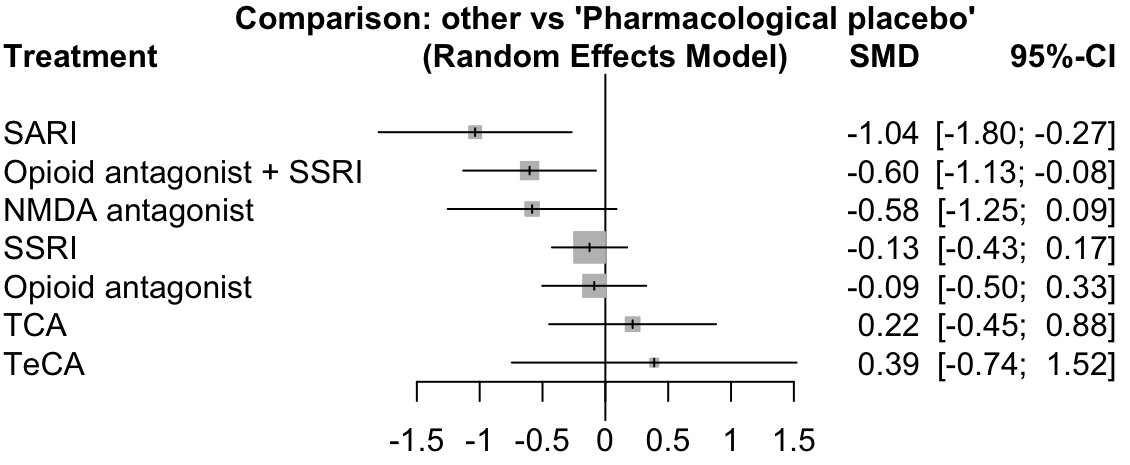


**eFigure 7.** Forest Plot of Effect Estimates (Active Pharmacological Interventions versus Pharmacological Placebo) on Functional Status at Post-Intervention


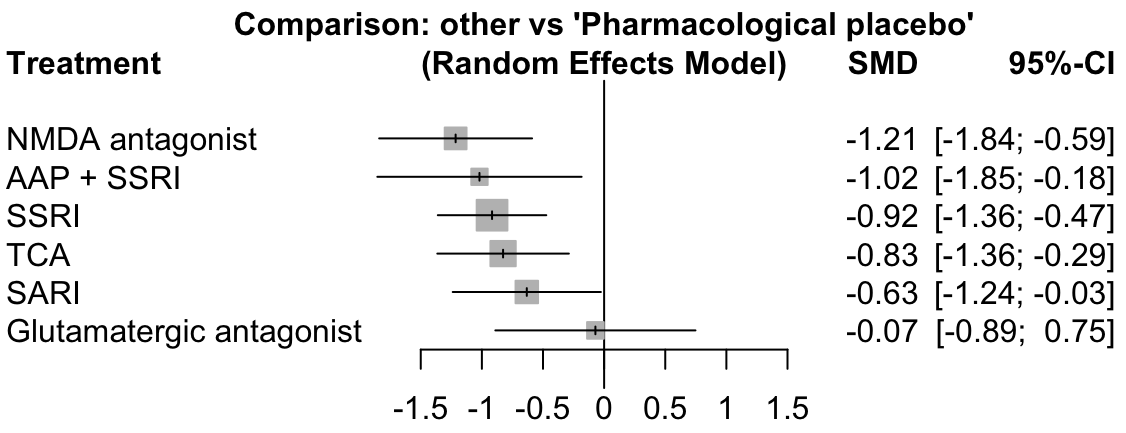


**eFigure 8.** Forest Plot of Effect Estimates (Active Pharmacological Interventions versus Pharmacological Placebo) on Adverse Events at Post-Intervention


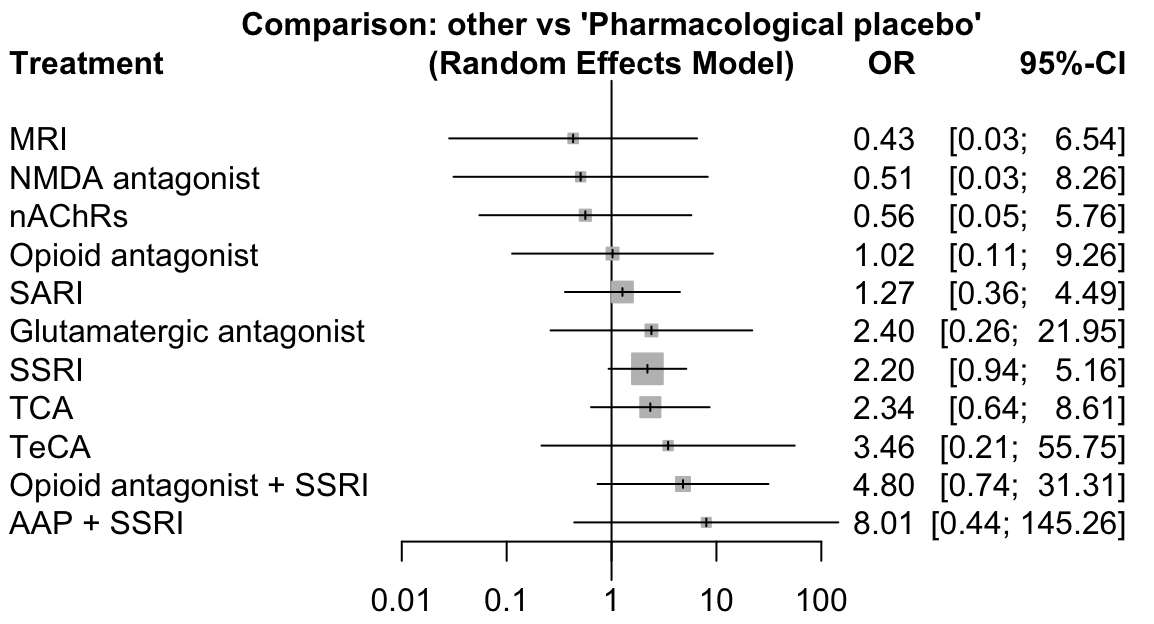


**eFigure 9.** Forest Plot of Effect Estimates (Active Pharmacological Interventions versus Pharmacological Placebo) on Serious Adverse Events at Post-Intervention


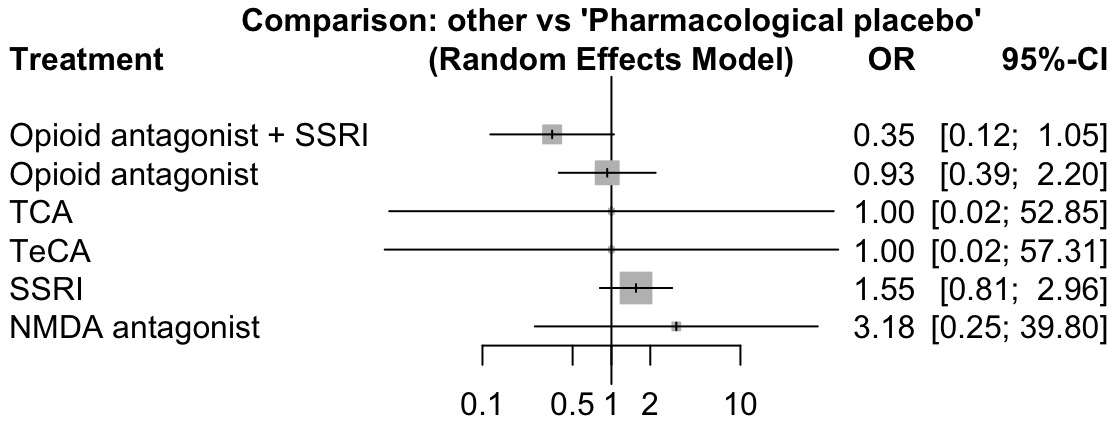


**eFigure 10.** Pharmacologic Network Geometry of Remission from Depression

**
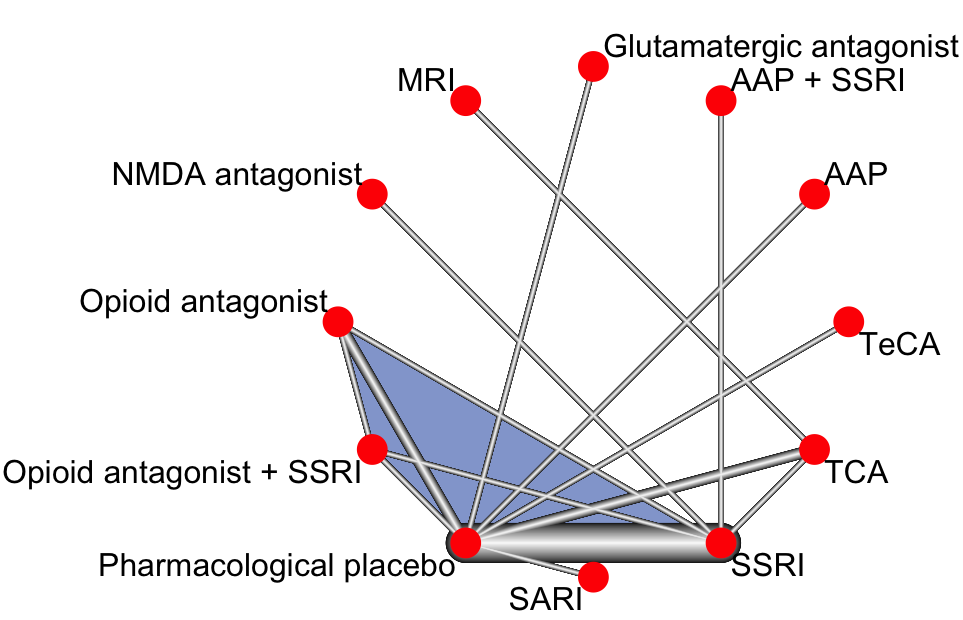
**

**eFigure 11.** Pharmacologic Network Geometry of Remission from Alcohol


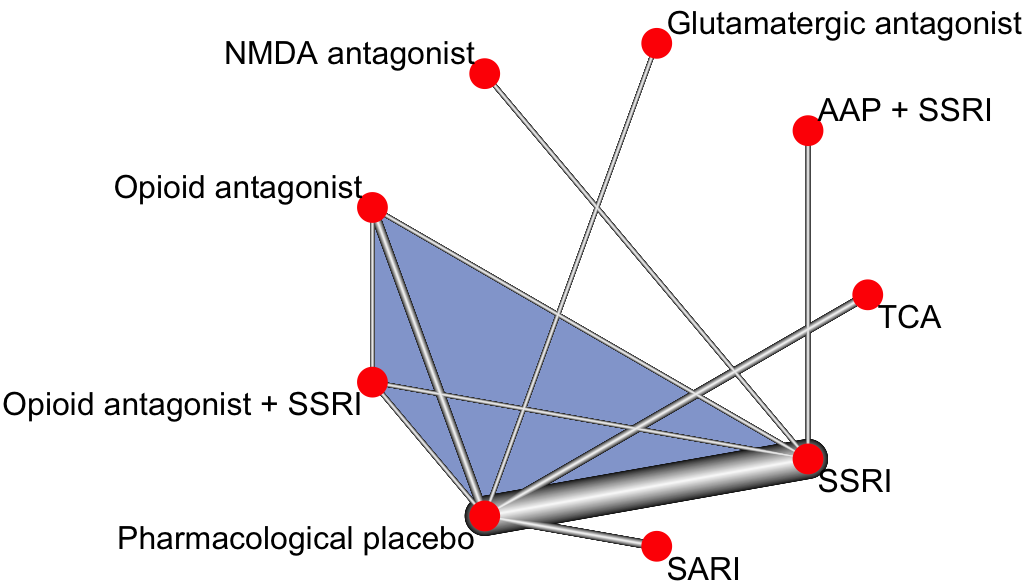


**eFigure 12.** Psychological Network Geometry of Remission from Alcohol

**
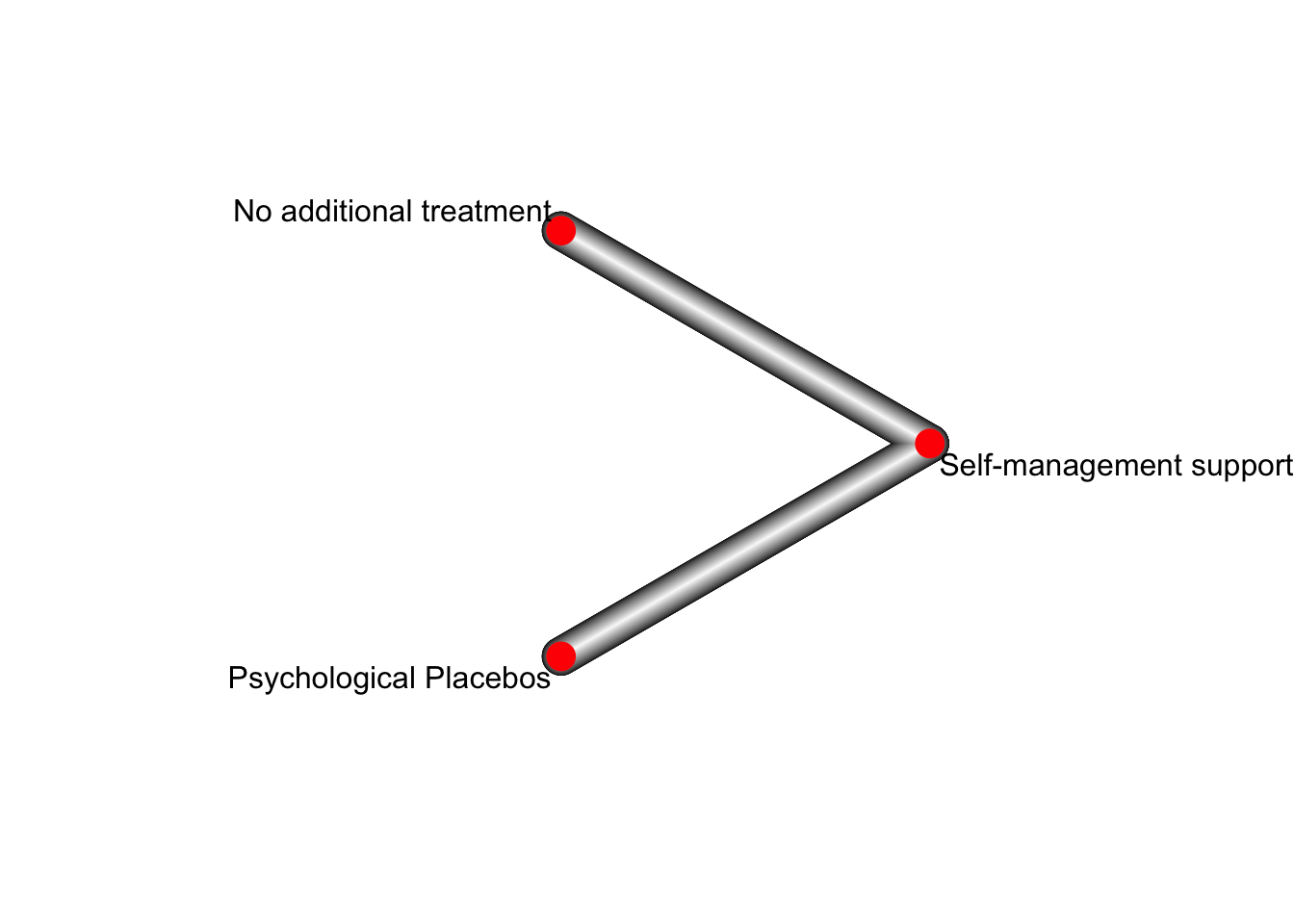
**

**eFigure 13.** Pharmacologic Network Geometry of Depressive Symptoms


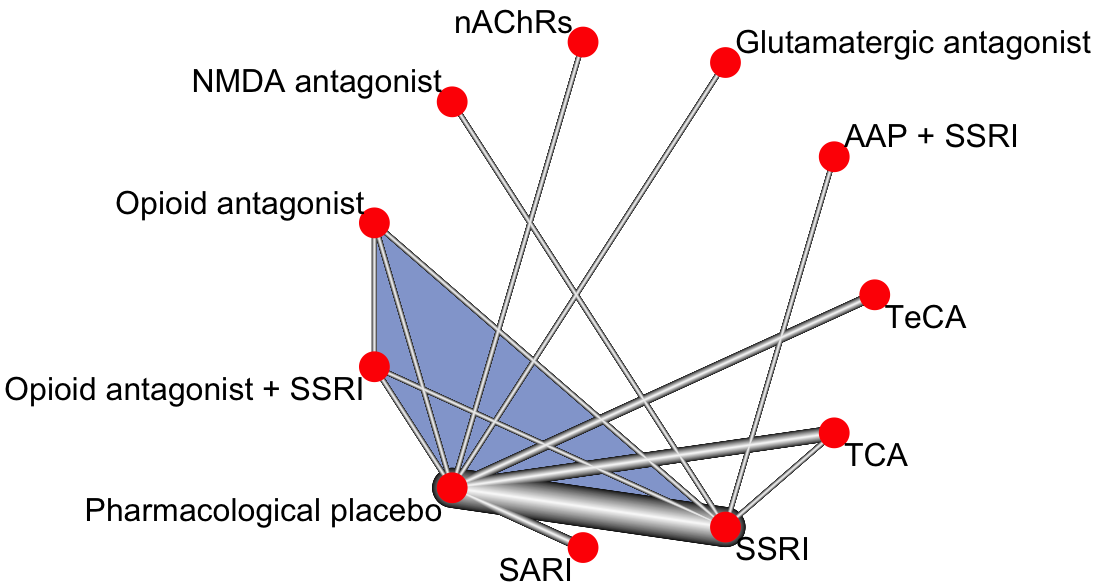


**eFigure 14.** Psychological Network Geometry of Depressive Symptoms


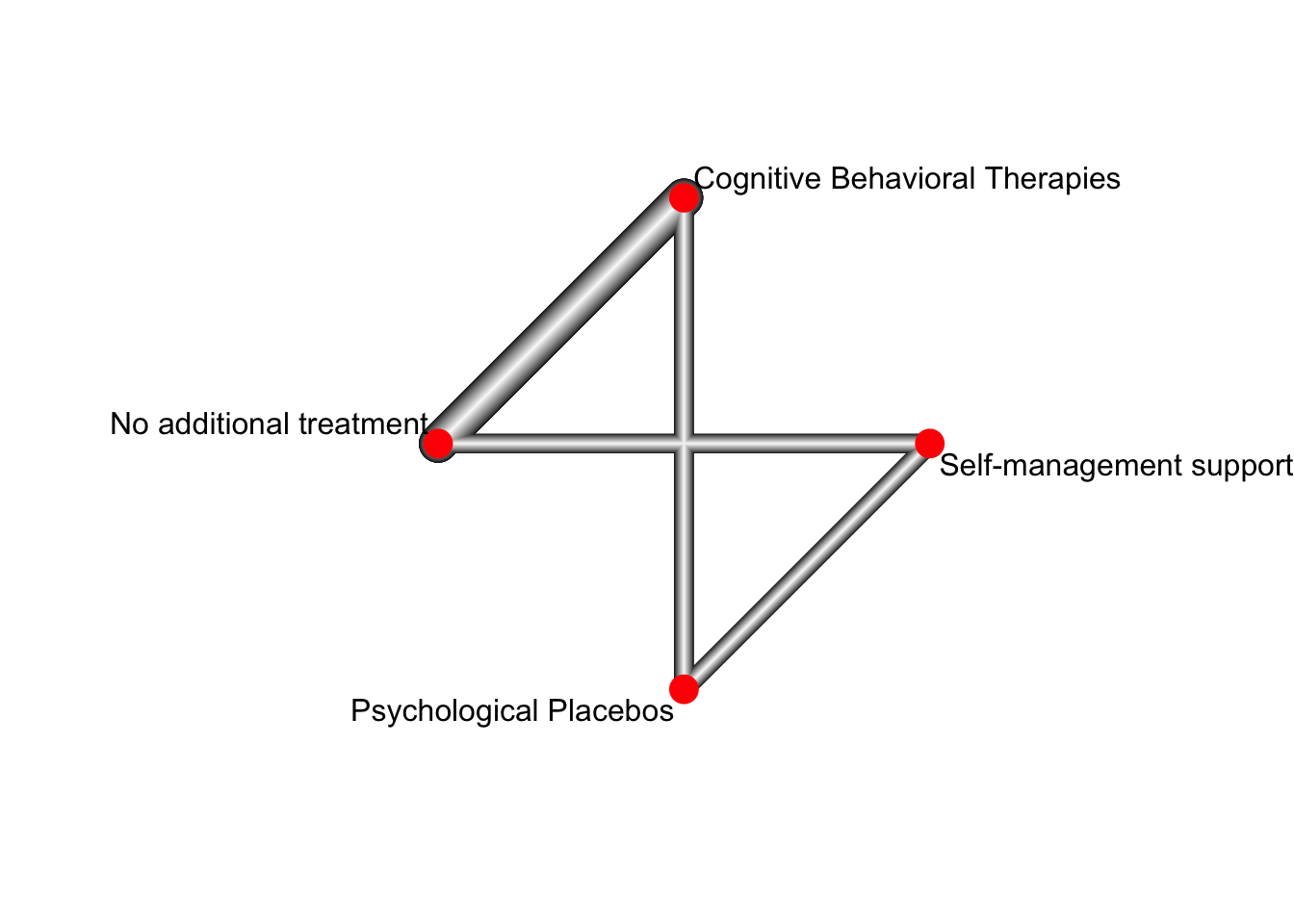


**eFigure 15.** Pharmacologic Network Geometry of Alcohol Use


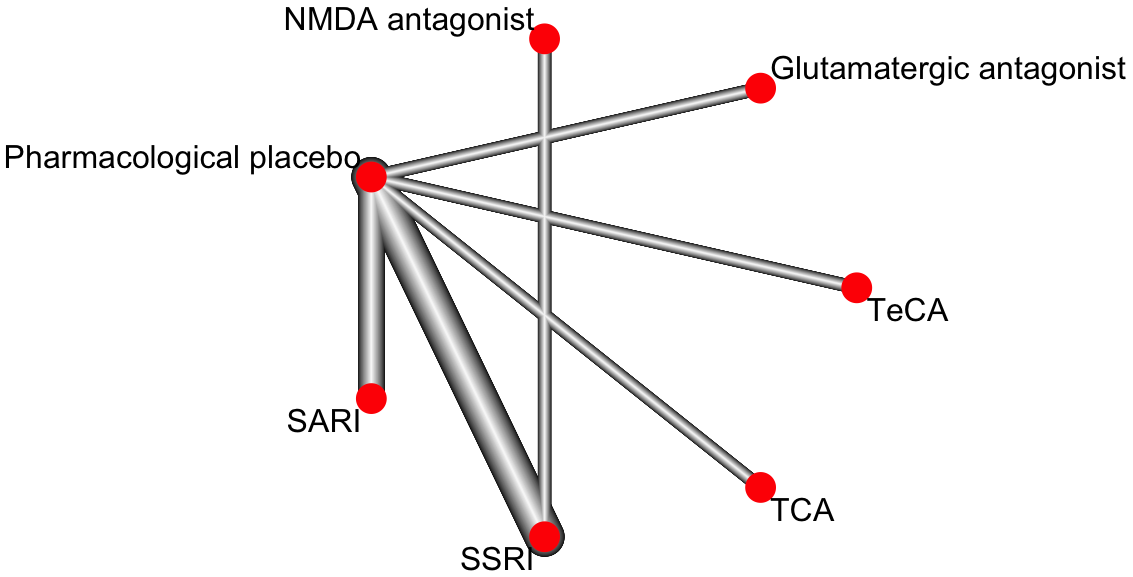


**eFigure 16.** Psychological Network Geometry of Alcohol Use

**
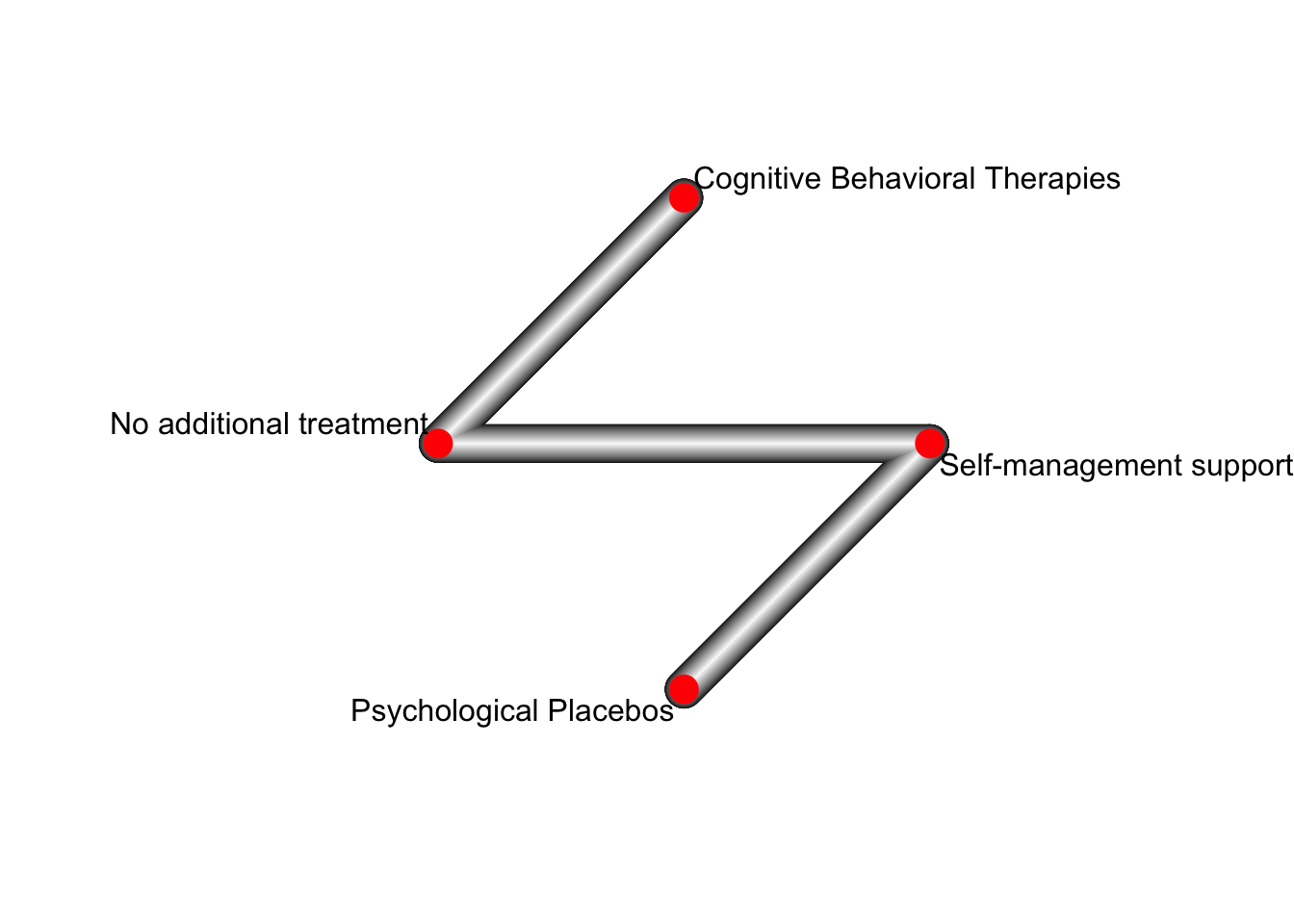
**

**eFigure 17.** Pharmacologic Network Geometry of Heavy Drinking


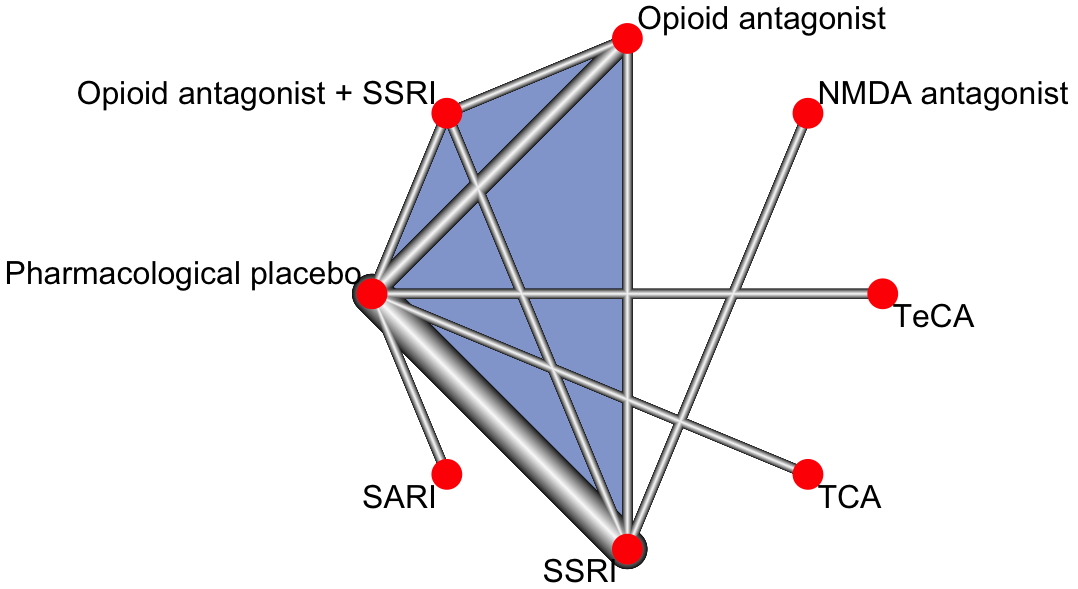


**eFigure 18.** Pharmacologic Network Geometry of Withdrawal/Craving Symptoms


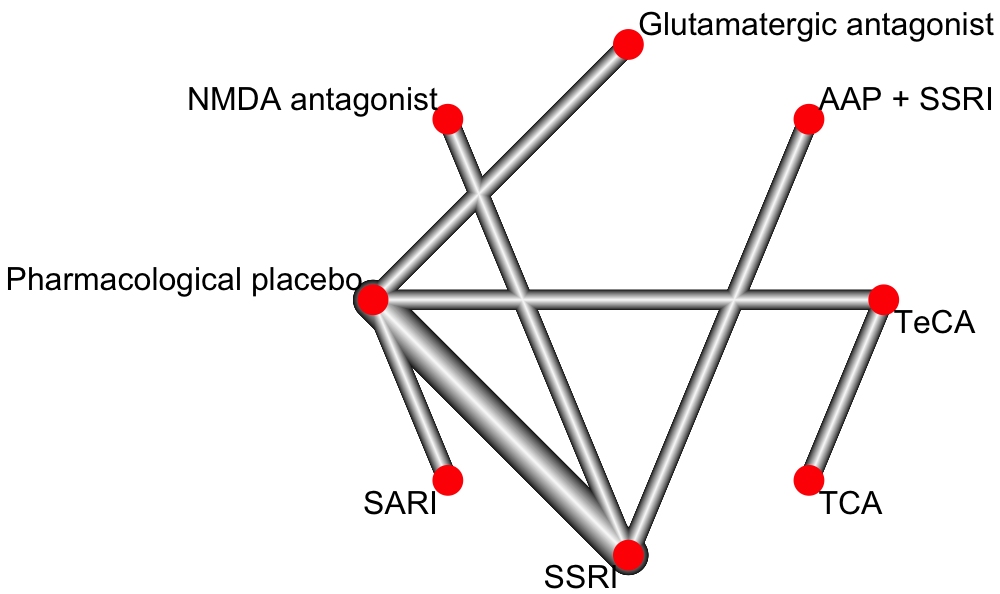


**eFigure 19.** Psychological Network Geometry of Withdrawal/Craving Symptoms

**
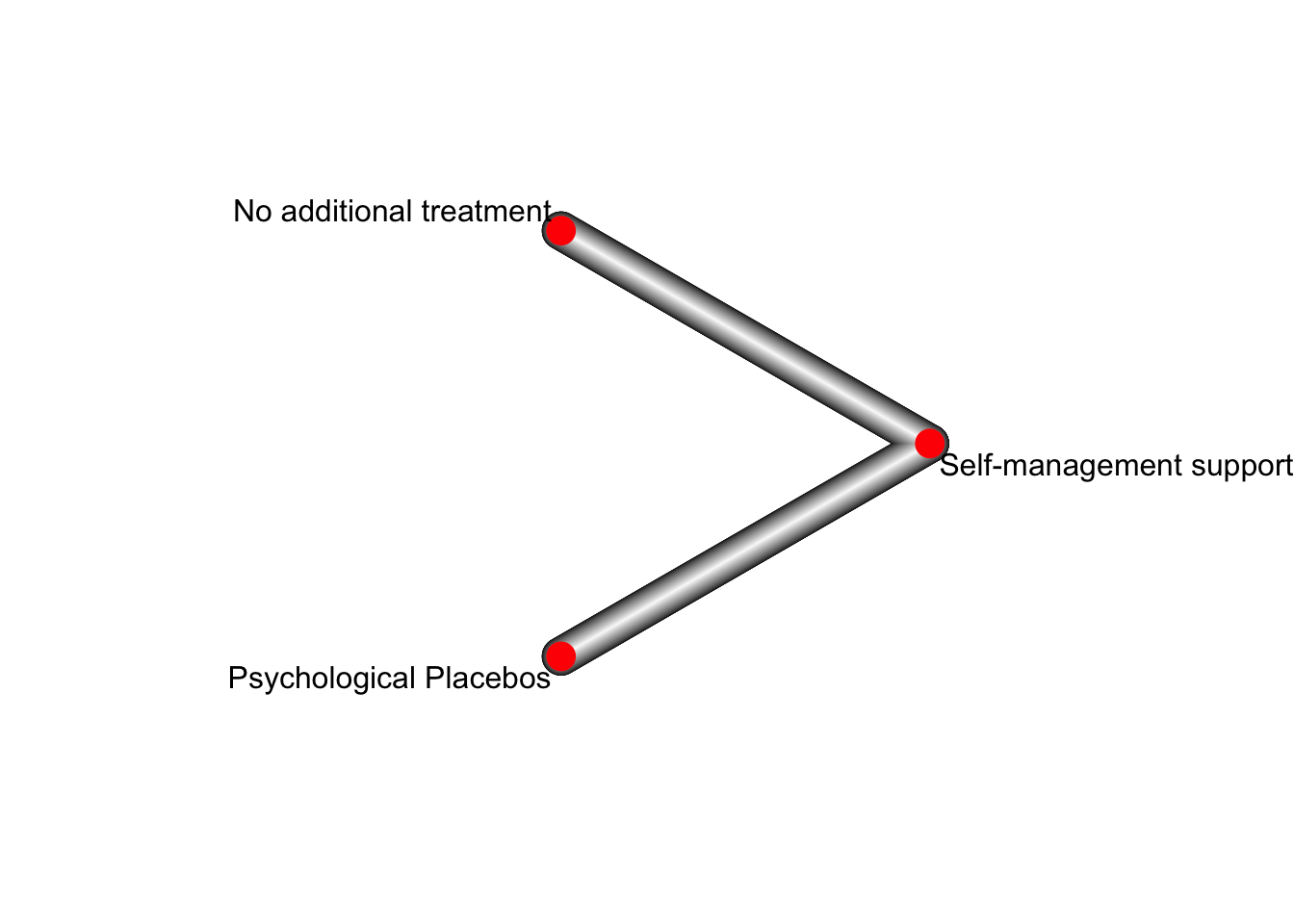
**

**eFigure 20.** Pharmacologic Network Geometry of Functional Status


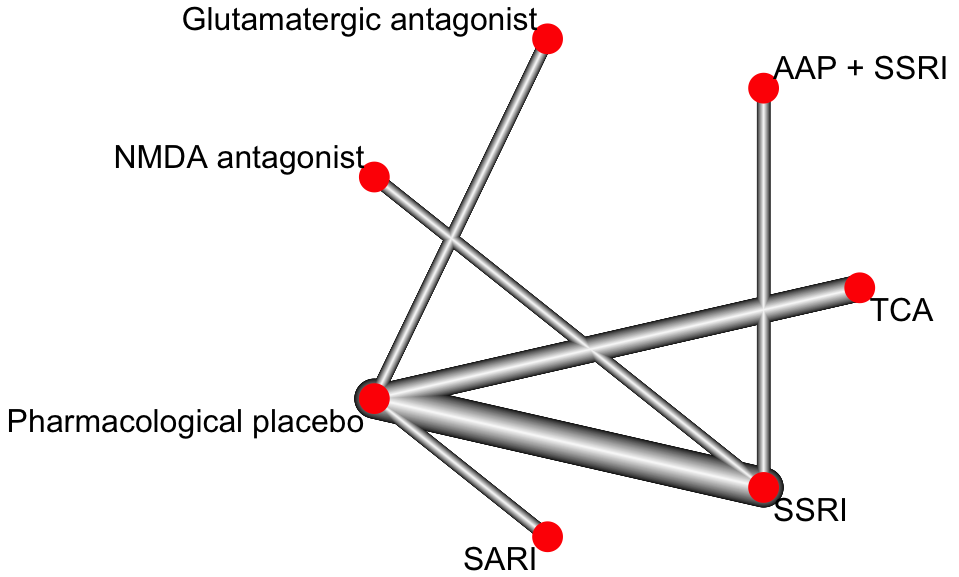


**eFigure 21.** Pharmacologic Network Geometry of Adverse Events


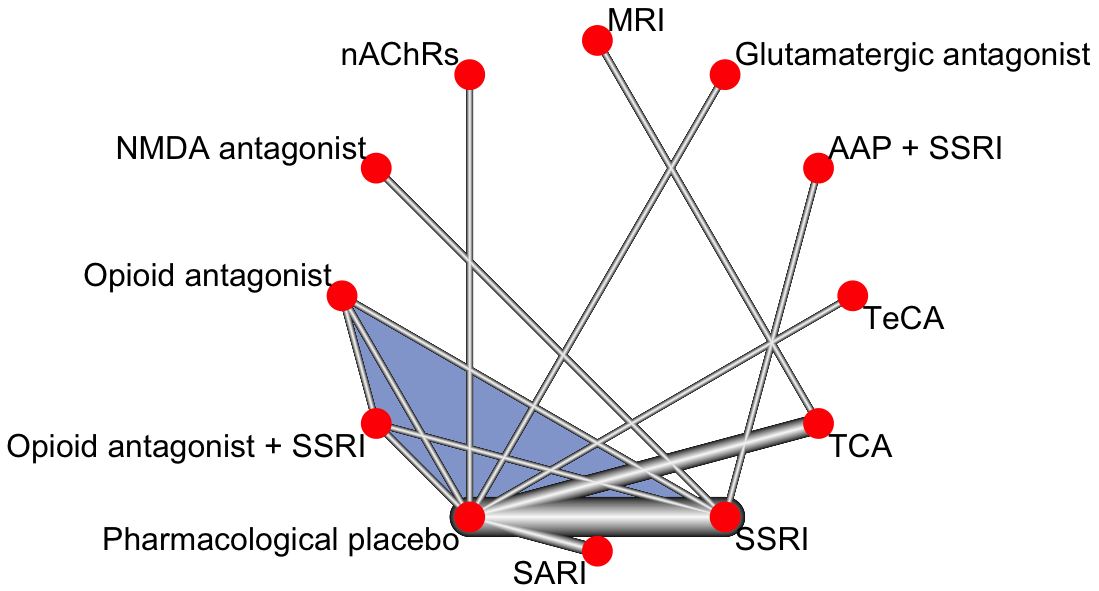


**eFigure 22.** Pharmacologic Network Geometry of Serious Adverse Events


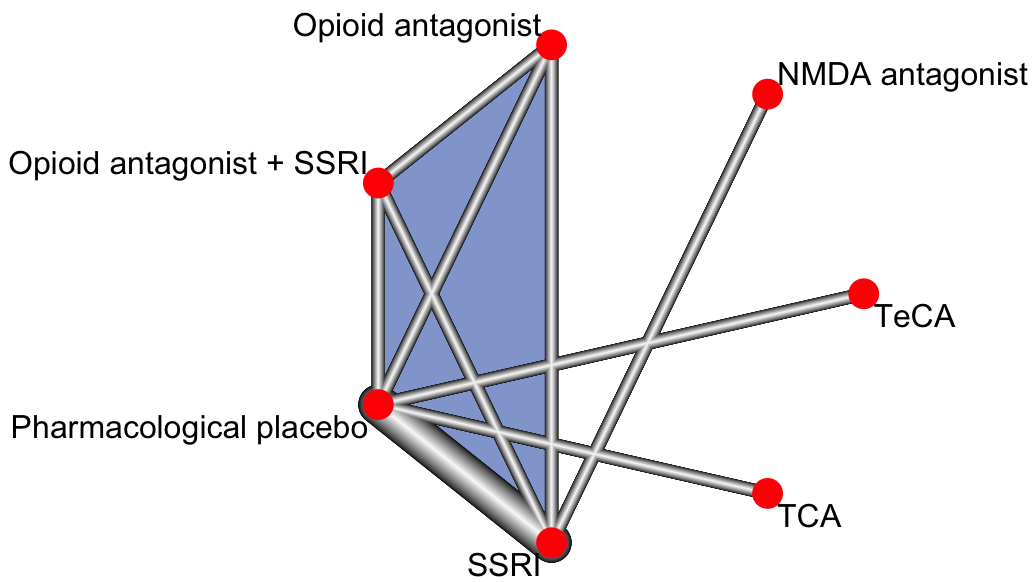


**eReferences**

1. Hutton B, Salanti G, Caldwell DM, Chaimani A, Schmid CH, Cameron C, et al. The PRISMA Extension Statement for reporting of systematic reviews incorporating network meta-analyses of health care interventions: checklist and explanations. Annals of Internal Medicine. 2015;162(11):777-84.

2. Moher D, Shamseer L, Clarke M, Ghersi D, Liberati A, Petticrew M, et al. Preferred Reporting Items for Systematic Review and Meta-Analysis Protocols (PRISMA-P) 2015 statement. Systematic Reviews. 2015;4(1):1.

3. Shamseer L, Moher D, Clarke M, Ghersi D, Liberati A, Petticrew M, et al. Preferred Reporting Items for Systematic Review and Meta-Analysis Protocols (PRISMA-P) 2015: elaboration and explanation. BMJ. 2015;349:g7647.

4. Higgins JPT, Lasserson T, Chandler J, Tovey D, Churchill R. Methodological Expectations of Cochrane Intervention Reviews. London: Cochrane; 2016.

5. Baker AL, Thornton LK, Hiles S, Hides L, Lubman DI. Psychological interventions for alcohol misuse among people with co-occurring depression or anxiety disorders: a systematic review. Journal of Affective Disorders. 2012;139:217-29.

6. DeVido JJ, Weiss RD. Treatment of the depressed alcoholic patient. Current Psychiatry Reports. 2012;14(6):610-8.

7. Foulds JA, Adamson SJ, Boden JM, Williman JA, Mulder RT. Depression in patients with alcohol use disorders: systematic review and meta-analysis of outcomes for independent and substance-induced disorders. Journal of Affective Disorders. 2015;185:47-59.

8. Hobbs JD, Kushner MG, Lee SS, Reardon SM, Maurer EW. Meta‐analysis of supplemental treatment for depressive and anxiety disorders in patients being treated for alcohol dependence. American Journal on Addictions. 2011;20(4):319-29.

9. Iovieno N, Tedeschini E, Bentley KH, Evins AE, Papakostas GI. Antidepressants for major depressive disorder and dysthymic disorder in patients with comorbid alcohol use disorders: a meta-analysis of placebo-controlled randomized trials. Journal of Clinical Psychiatry. 2011;72(8):1144-51.

10. Nunes EV, Levin FR. Treatment of depression in patients with alcohol or other drug dependence: a meta-analysis. JAMA. 2004;291(15):1887-96.

11. Riper H, Andersson G, Hunter SB, Wit J, Berking M, Cuijpers P. Treatment of comorbid alcohol use disorders and depression with cognitive‐behavioural therapy and motivational interviewing: a meta‐analysis. Addiction. 2014;109(3):394-406.

12. Grant ES, Calderbank-Batista T. Network meta-analysis for complex social interventions: Problems and potential. Journal of the Society for Social Work and Research. 2013;4(4):406-20.

13. Klimas J, Tobin H, Field CA, O'Gorman CS, Glynn LG, Keenan E, et al. Psychosocial interventions to reduce alcohol consumption in concurrent problem alcohol and illicit drug users. Cochrane Database of Systematic Reviews. 2014;(12):CD009269.

14. Agyapong VI, Ahern S, McLoughlin DM, Farren CK. Supportive text messaging for depression and comorbid alcohol use disorder: single-blind randomised trial. J Affect Disord. 2012;141(2-3):168-76. Epub 2012/04/03. doi: 10.1016/j.jad.2012.02.040. PubMed PMID: 22464008.

15. Ferri M, Amato L, Davoli M. Alcoholics Anonymous and other 12-step programmes for alcohol dependence. Cochrane Database of Systematic Reviews. 2006;(3):CD005032.

16. James A, Yavchitz A, Ravaud P, Boutron I. Node making process in network meta-analysis of non-pharmacological treatment are poorly reported. Journal of Clinical Epidemiology. 2018;97:95-102. doi: 10.1016/j.jclinepi.2017.11.018.

17. Welch V, Petticrew M, Tugwell P, Moher D, O'Neill J, Waters E, et al. PRISMA-Equity 2012 Extension: Reporting Guidelines for Systematic Reviews with a Focus on Health Equity. PLoS Medicine. 2012;9(10):e1001333.

18. Welch V, Petticrew M, Petkovic J, Moher D, Waters E, White H, et al. Extending the PRISMA statement to equity-focused systematic reviews (PRISMA-E 2012): explanation and elaboration. International Journal for Equity in Health. 2015;14(1):92.

19. Hoffmann TC, Glasziou P, Boutron I, Milne R, Perera R, Moher D, et al. Better reporting of interventions: Template for intervention description and replication (TIDieR) checklist and guide. BMJ. 2014;348:g1687.

20. Zarin DA, Tse T, Williams RJ, Califf RM, Ide NC. The ClinicalTrials. gov results database—update and key issues. New England Journal of Medicine. 2011;364(9):852-60.

21. Damschroder LJ, Aron DC, Keith RE, Kirsh SR, Alexander JA, Lowery JC. Fostering implementation of health services research findings into practice: a consolidated framework for advancing implementation science. Implementation Science. 2009;4(1):50.

22. Higgins JPT, Sterne JAC, Savović J, Page MJ, Hróbjartsson A, Boutron I, et al. A revised tool for assessing risk of bias in randomized trials. Cochrane Database of Systematic Reviews. 2016;10(Suppl 1):29-31. doi: dx.doi.org/10.1002/14651858.CD201601.

23. Higgins J, Altman D, Gøtzsche P, Jüni P, Moher D, Oxman A, et al. The Cochrane Collaboration’s tool for assessing risk of bias in randomised trials. BMJ. 2011;343:d5928.

24. Salanti G. Indirect and mixed-treatment comparison, network, or multiple-treatments meta-analysis: many names, many benefits, many concerns for the next generation evidence synthesis tool. Research Synthesis Methods. 2012;3(2):80-97.

25. Rücker G, Schwarzer G, Krahn U, König J, Schwarzer MG. Package ‘netmeta’: Network Meta-Analysis using Frequentist Methods 2019. Available from: <https://cran.r-project.org/web/packages/netmeta/netmeta.pdf>.

26. Salanti G, Ades AE, Ioannidis JP. Graphical methods and numerical summaries for present- ing results from multiple-treatment meta-analysis: an overview and tutorial. Journal of Clinical Epidemiology. 2011;64(2):163-71.

27. Rücker G, Schwarzer G. Ranking treatments in frequentist network meta-analysis works without resampling methods. BMC Medical Research Methodology. 2015;15(1):58.

28. Rücker G. Network meta-analysis, electrical networks and graph theory. Research Synthesis Methods. 2012;3:312-24.

29. Rücker G, Schwarzer G. Reduce dimension or reduce weights? Comparing two approaches to multi-arm studies in network meta-analysis. Statistics in Medicine. 2014;33:4353-69.

30. Jackson D, White IR, Riley RD. Quantifying the impact of between-study heterogeneity in multivariate meta-analyses. Statistics in Medicine. 2012;31(29):3805-20.

31. Dias S, Welton NJ, Caldwell DM, Ades AE. Checking consistency in mixed treatment comparison meta-analysis. Statistics in Medicine. 2010;29:932-44.

32. Krahn U, Binder H, König J. A graphical tool for locating inconsistency in network meta-analyses. BMC Medical Research Methodology. 2013;13(1):35.

33. Brignardello-Petersen R, Bonner A, Alexander PE, Siemieniuk RA, Furukawa TA, Rochwerg B, et al. Advances in the GRADE approach to rate the certainty in estimates from a network meta-analysis. Journal of Clinical Epidemiology. 2018;93:36-44.

34. Guyatt GH, Oxman AD, Vist GE, Kunz R, Falck-Ytter Y, Alonso-Coello P, et al. GRADE: an emerging consensus on rating quality of evidence and strength of recommendations. BMJ. 2008;336(7650):924.

35. Puhan MA, Schünemann HJ, Murad MH, Li T, Brignardello-Petersen R, Singh JA, et al. A GRADE Working Group approach for rating the quality of treatment effect estimates from network meta-analysis. BMJ. 2014;349:g5630.

36. Salanti G, Del Giovane C, Chaimani A, Caldwell DM, Higgins JP. Evaluating the quality of evidence from a network meta-analysis. PLoS One. 2014;9(7):e99682.

37. Guyatt GH, Oxman AD, Vist G, Kunz R, Brozek J, Alonso-Coello P, et al. GRADE guidelines: 4. Rating the quality of evidence—study limitations (risk of bias). Journal of Clinical Epidemiology. 2011;64(4):407-15.

38. Guyatt GH, Oxman AD, Kunz R, Woodcock J, Brozek J, Helfand M, et al. GRADE guidelines: 8. Rating the quality of evidence—indirectness. Journal of Clinical Epidemiology. 2011;64(12):1303-10.

39. Guyatt GH, Oxman AD, Kunz R, Woodcock J, Brozek J, Helfand M, et al. GRADE guidelines: 7. Rating the quality of evidence—inconsistency. Journal of Clinical Epidemiology. 2011;64(12):1294-302.

40. Guyatt GH, Oxman AD, Kunz R, Brozek J, Alonso-Coello P, Rind D, et al. GRADE guidelines 6. Rating the quality of evidence—imprecision. Journal of Clinical Epidemiology. 2011;64(12):1283-93.

41. Guyatt GH, Oxman AD, Montori V, Vist G, Kunz R, Brozek J, et al. GRADE guidelines: 5. Rating the quality of evidence—publication bias. Journal of Clinical Epidemiology. 2011;64(12):1277-82.

42. Hultcrantz M, Rind D, Akl EA, Treweek S, Mustafa RA, Iorio A, et al. The GRADE Working Group clarifies the construct of certainty of evidence. Journal of Clinical Epidemiology. 2017;87:4-13.

43. American Psychiatric Association. Diagnostic and statistical manual of mental disorders. 5th ed. Washington, DC: Author; 2013.

44. World Health Organization. The ICD-10 classification of mental and behavioural disorders: Clinical descriptions and diagnostic guidelines. Geneva: Author; 1992.

45. American Psychiatric Association. Diagnostic and statistical manual of mental disorders. 4th ed. Washington, DC: Author; 2000.
