# Supplementary material for: Clinical interventions for adults with comorbid alcohol use and depressive disorders: A systematic review and network meta-analysis
Source: PLoS Med. 2021 Oct 8;18(10):e1003822. doi: 10.1371/journal.pmed.1003822 (PMC8535380; doi:10.1371/journal.pmed.1003822)
Supplement: Attachment — Submitted filename: Alcohol Depression NMA Revision 2 - Response to Reviewers.docx [file pmed.1003822.s005.docx]

**Requests from the editors**

1. Please update the search to a point in the past 3 months, say.

- We have updated the search through December 1, 2020. Through this update search, we have identified one additional paper for an already included study (Adamson 2015), one additional included study (O'Reily 2019), and eight additional studies that are ongoing/awaiting assessment. We have revised all aspects of the manuscript (including the flow diagram in Figure 1) to reflect the update.

2. Please add a sentence to your abstract to summarize where the constituent studies were done and the timeframe, and the median study size.

- We have added the following sentence to the abstract: “Studies were published from 1971 to 2019, conducted in 13 countries, and had a median sample size of 50 participants (range: 14-to-350 participants).”

3. In the abstract and elsewhere, you discuss adverse events in patients receiving SSRIs, with a p=0.07. In that this finding does not appear to be statistically significant, please explain why "moderate confidence" is ascribed to both this finding and the apparent benefit of SSRIs for functional status (p<0.001). Is the word "significantly" at line 331 appropriate?

- Thank you for this important question. According to the GRADE approach, we rated adverse events of SSRIs down one level (i.e., from “high” to “moderate” confidence) because of the wide confidence interval crossing the line of no effect (i.e., p > 0.05). Per our methods, we did not downgrade two levels for imprecision: while the 95% Confidence Interval included the null effect (“serious” imprecision), it did not include the threshold for a meaningful reduction in the likelihood of experiencing an adverse event (“very serious” imprecision). In addition, we did not find sufficient reason to downgrade due to study limitations, indirectness, inconsistency, publication bias, intransitivity, or incoherence. Consequently, we interpret the effect estimate indicting that patients receiving SRRIs had a greater risk of experiencing an adverse event, though with moderate confidence. In other words, using language from the GRADE approach, patients receiving SSRIs likely had a greater risk of experiencing an adverse event compared to patients receiving pharmacological placebos. In contrast, we have “moderate” confidence in the estimate of SSRIs on functional status due to a different domain from the GRADE approach (i.e., the study limitation of selective outcome reporting, rather than imprecision).
  - The explanation for moderate confidence in adverse events now reads as follows: “We have moderate confidence that patients receiving SSRIs were more likely to experience an adverse event than patients receiving pharmacological placebos (OR = 2.20; 95% CI, 0.94 to 5.16; 6 RCTs; p = 0.07). We downgraded confidence from “high” to “moderate” because of a wide confidence interval that included the null effect (i.e., “serious” imprecision). However, we did not downgrade two levels (i.e., “very serious” imprecision), because the confidence interval did not include the threshold for a meaningful reduction in the likelihood of experiencing an adverse event. In addition, we did not find sufficient reason to downgrade due to study limitations, indirectness, inconsistency, publication bias, intransitivity, or incoherence.”
  - The explanation for moderate confidence in functional status now reads as follows: “We have moderate confidence in an estimate of at least a small beneficial effect at postintervention of SSRIs over placebos (SMD = -0.92; 95% CI, -1.36 to -0.47; 3 RCTs; p < 0.001); we downgraded confidence from “high” to “moderate” because of a risk of selective outcome reporting (i.e., “serious” limitations of included studies).”
- We agree that "significantly" at line 331 in the previous version was inappropriate, and we have revised to: “However, we also found SSRIs to have a higher risk of adverse events (including serious adverse events). Using language from the GRADE approach, patients receiving SSRIs likely had a greater risk of experiencing an adverse event compared to patients receiving pharmacological placebos (moderate confidence), and they may have had a greater risk of experiencing a serious adverse event (low confidence).”

4. In the final sentence of the "Methods and Findings" subsection of your abstract, please limit this to 2-3 study limitations.

- We have revised the limitations in the abstract as follows: “Limitations include the sparsity of evidence on intervention effects over the long-term, risks of attrition bias, and heterogeneous definitions of adverse events in the evidence base.”

5. Please use "White" rather than "Caucasian", e.g., at line 199.

- We have removed “Caucasian” as suggested.

6. Do you see any issues in comparing data from RCTs involving drugs, often placebo controlled, with those from RCTs involving cognitive therapies, where there is often no control for attention? We might have missed this in the current paper, but if not you may wish to address this in the discussion section.

- We thank the editor for this question. To clarify, as we did not identify any RCTs comparing a pharmacological intervention to a psychological intervention, we have two separate networks, and therefore do not draw any comparisons between pharmacological and psychological interventions. We have added the following note to the Discussion to address this issue: “Furthermore, as we did not identify any RCTs comparing a pharmacological to a psychological intervention, we had analyze these families of interventions in separate networks, thereby preventing us from drawing any comparisons between (classes of) pharmacological and psychological interventions. Consequently, we caution readers in any such comparisons they may make using the results of this review.”

7. Please do not use italics for emphasis.

- We believe that we have removed all uses of italics for emphasis in the manuscript, though we do apologize if we have missed any instances and authorize the copy editor to make such changes should the manuscript be accepted.

8. Throughout the paper, please remove spaces from within the square brackets for reference call-outs (e.g., "... depressive disorder [6,14].".

- We have revised as requested.

9. Please revisit "Author" in references 4 & 5, which should perhaps be removed.

- We have removed as requested.

10. Please adapt the header for figure 1 to "Study flow diagram" or similar.

- We have revised as requested.

**Reviewer 2**

1. The authors have done a good job responding to my comments and comments from other reviewers. However, I remain concerned about the presentation of the results, particularly in the abstract.

- We thank the reviewer for the kind words about our previous revision, and we hope to have addressed remaining concerns in this revision.

2. The results of the primary outcomes (remission from depression and remission from alcohol use) should explicitly be described in the abstract, in order to avoid any misinterpretation of the results and 'spin'.

- We sincerely appreciate the reviewer’s attention to “spin”, as it certainly was not our intention. We had focused on outcomes with “low” or higher confidence per the GRADE approach, though we agree that the primary outcomes should be explicitly mentioned so as to avoid any misinterpretation. We have revised as follows:
  - Abstract—Methods and Findings: “We have very low confidence in all estimates of intervention effects on our primary outcomes (i.e., remission from depression and remission from alcohol use).”
  - Abstract—Conclusions: “We are very uncertain about the existence (or not) of any nonnull effects for our primary outcomes of remission from depression and remission from alcohol use.”
  - Author Summary— What did the researchers do and find: “We have very low confidence in all estimates of intervention effects on our primary outcomes (i.e., remission from depression and remission from alcohol use).”
  - Author Summary— What do these findings mean: “For policy and practice, we are very uncertain about the existence (or not) of any nonnull effects for our primary outcomes of remission from depression and remission from alcohol use. The available evidence does suggest potentially actionable benefits at postintervention of CBTs for depressive symptoms and alcohol use, TCAs for depressive symptoms, and SSRIs for alcohol use and functional status-though SSRIs also likely have higher risks of adverse events (including serious adverse events).”
  - Discussion: “The available body of evidence on treatments for adults with both an alcohol use and depressive disorder includes 14 pharmacological interventions and four psychological interventions. These interventions represent a fraction of the interventions discussed and recommended in clinical practice guidelines for either alcohol use or depressive disorders [7,8]. Moreover, we have very low confidence in all estimates of intervention effects on our primary outcomes (i.e., remission from depression and remission from alcohol use).”
  - Conclusions: “Those charged with developing guidelines, providing recommendations for health systems, and treating patients may be interested in using these findings to inform policy and practice. We are very uncertain about the existence (or not) of any nonnull effects for our primary outcomes of remission from depression and remission from alcohol use. The available evidence does suggest potentially actionable benefits for patients with both an AUD and a depressive disorder at postintervention of CBTs for depressive symptoms and alcohol use, TCAs for depressive symptoms, and SSRIs for alcohol use and functional status—though SSRIs also have higher risks of adverse events (including serious adverse events).”

3. The fact that this review found no high grade evidence for the use of pharmacological and psychological interventions in patients with co-occurring AUDs and depressive disorders should also be highlighted. In my view, it is the key message to policy makers that should be put forward.

- We thank the reviewer for highlighting this important takeaway as well. We have revised the manuscript as follows:
  - Abstract—Methods and Findings: “We have very low confidence in all other effect estimates, and we did not have high confidence in any effect estimates.”
  - Abstract—Conclusions: “As we did not have high confidence in any outcomes, additional studies are warranted to provide more conclusive evidence.”
  - Author Summary— What did the researchers do and find: “We have very low confidence in all other effect estimates, and we did not have high confidence in any effect estimates.”
  - Author Summary— What do these findings mean: “We did not have high confidence in any effect estimates, and we have very low confidence in the vast majority of estimates of intervention effects across all outcomes.”
  - Discussion: “Moreover, we have very low confidence in all estimates of intervention effects on our primary outcomes (i.e., remission from depression and remission from alcohol use). We also did not have high confidence in any effect estimates, and we have very low confidence in the vast majority of estimates of intervention effects across all outcomes. We are confident only in estimates at postintervention about the benefits of CBTs (on depressive symptoms and alcohol use), SSRIs (on functional status and alcohol use), and TCAs (on depressive symptoms) to be sufficient enough to warrant their consideration for policy and practice.”
  - Conclusions: “We are very uncertain about the existence (or not) of any nonnull effects for our primary outcomes of remission from depression and remission from alcohol use. We also did not have high confidence in any effect estimates, and we have very low confidence in the vast majority of estimates of intervention effects across all outcomes. The available evidence does suggest potentially actionable benefits for patients with both an AUD and a depressive disorder at postintervention of CBTs for depressive symptoms and alcohol use, TCAs for depressive symptoms, and SSRIs for alcohol use and functional status—though SSRIs also have higher risks of adverse events (including serious adverse events).”

4. Some of the limitations of the study do not need to be highlighted in the abstract, such as the lack of contact with some trial authors for missing information and data, or the use of SMDs rather than mean differences. Conversely, crucial limitations preventing a clear interpretation of the data are missing, namely the absence of demonstrated treatment effect over the long-term, and an attrition bias that cannot be ruled out (half of the studies had an unclear or high risk of bias related to completeness of outcome data at post intervention).

- Per the reviewer’s and the editor’s suggestions, we have revised the limitations in the abstract as follows: “Limitations include the sparsity of evidence on intervention effects over the long-term, risks of attrition bias, and heterogeneous definitions of adverse events in the evidence base.”

**Reviewer 3**

1. I confine my remarks to statistical aspects of this paper.  In general, these were very thorough and well done and I have only some minor issues to resolve before I can recommend publication.

- We thank the reviewer for the kind words about the thoroughness of our work. We hope to have resolved the remaining minor issues in this revision.

2. The biggest one is defining networks.  Maybe I missed it (but I did search the document for the term "Network" and I did look at S1) but I couldn't figure out how studies were assigned to networks or what they were for.

- We thank the reviewer for this comment. We provide additional information about networks as follows:
  - Background: “To achieve this objective, we performed a network meta-analysis (NMA). An NMA combines both direct and indirect comparisons of intervention effects, obtaining an effect estimate for each possible pair of interventions (including those that have not been directly compared). Consequently, identifying and synthesizing evidence from the entire network of evidence enables a more comprehensive understanding of the comparative effectiveness of interventions for a given population and outcome. As such, it is a powerful research tool to assist patients, providers, and policymakers to make informed decisions about which intervention is most likely to improve health care at the individual and population levels.”
  - Methods— Identification and Selection of Studies: “A crucial aspect of NMAs involves visualizing the interventions that have been evaluated for a population of interest as forming a network in which the interventions are represented by dots (or “nodes”) and comparisons between interventions are represented by lines (or “edges”) in a diagram [33]. After completing the search but before extracting and analyzing outcome data, we assigned identified interventions to nodes in our network via consensus among the review team and external advisers, using a pre-registered list of intervention nodes (see S1 Text) as a guide [24].”

3. In addition, I'd prefer a little less emphasis on tests of significance in the heterogeneity analysis. I like the use of heat maps and I think the authors should emphasize the effect size -- how different were the studies? -- rather than whether the differences were significant.

- We appreciate the author’s interest in the net heat maps to assess inconsistency in the network (i.e., incoherence). We do believe the tests of significance are an important tool to investigate incoherence in combination with net heat plots, which on their own may not serve as reliable methods for detecting incoherence (see e.g., <https://onlinelibrary.wiley.com/doi/full/10.1002/sim.8383>). When a net heat plot was possible for a given network (i.e., a closed loop in the network geometry), and we had more than “very low confidence” for an effect estimate in that network, we have revised the manuscript to provide more details on the interventions and their effect sizes. These criteria were met for the pharmacologic network on depressive symptoms: “We detected significant heterogeneity in the pharmacologic network globally (Q(12) = 20.82, p = 0.05), which was due to significant within-design heterogeneity in comparisons of placebos versus SSRIs (Q(6) = 15.38, p = 0.02) rather than inconsistency between designs (Q(2) = 3.53, p = 0.17). Hotspots of inconsistency were absent from (and direct versus indirect effect estimates were in the same direction in) the net heat plot investigating SSRIs versus placebo (OR from direct evidence = 0.11, OR from indirect evidence = 0.49), TCAs versus placebo (OR from direct evidence = 0.52, OR from indirect evidence = 0.11), and SSRIs versus TCAs (OR from direct evidence = 0.01, OR from indirect evidence = 0.41).” Further details about the heat maps and the differences in effect sizes can be found in S1-Appendix. As we believe that these further details do not influence the substantive conclusions of the review on the comparative effectiveness of interventions, we respectfully have elected not to add further detail (beyond that mentioned above for depressive symptoms) to the manuscript.
