# Supplementary material for: Clinical interventions for adults with comorbid alcohol use and depressive disorders: A systematic review and network meta-analysis
Source: PLoS Med. 2021 Oct 8;18(10):e1003822. doi: 10.1371/journal.pmed.1003822 (PMC8535380; doi:10.1371/journal.pmed.1003822)
Supplement: Attachment — Submitted filename: Alcohol Depression NMA Revision 3 - Response to Reviewers.docx [file pmed.1003822.s006.docx]

**Requests from the editors**

1. Please use the form "non-null" throughout.

- We have searched all instances of “nonnull” and have replaced with “non-null”.

2. Please abbreviate journal names in your reference list, including "PLoS ONE" and "PLoS Med.".

- We have abbreviated all journal names in our reference list using PubMed formatting.

3. Please re-label the attached checklist "S1_PRISMA_Checklist", and use this label in the text.

- We have re-labeled “S1 Table” as “S1_PRISMA_Checklist”, and used this label in the text.

4. Please confirm that no display items have been used in previous publications (in order to be published under a CC BY licence, they must be free of copyright).

- We confirm that we have created all figures ourselves and they may be published in this article using a CC BY license.
